# Complex Dynamics in Argyrodite Solid-State Ion Conductors

**DOI:** 10.1021/acs.chemmater.5c02939

**Published:** 2026-03-27

**Authors:** Austin M. Shotwell, Shelby L. Galinat, Annalise E. Maughan

**Affiliations:** † Department of Chemistry, 3557 Colorado School of Mines, Golden, Colorado 80401, United States; ‡ Materials Science Program, Colorado School of Mines, Golden, Colorado 80401, United States; § National Laboratory of the Rockies, Golden, Colorado 80401, United States

## Abstract

Argyrodites are a compositionally diverse family of materials that exhibit remarkable ion transport properties. While the average crystal structures of argyrodites have been extensively studied, ion transport in these materials is governed by a confluence of dynamic processes spanning the cation, anion, and polyanionic sublattices. This Perspective synthesizes recent advances in understanding the role of dynamics in structural behavior and ion transport properties. We examine the compositional and structural motifs that govern order–disorder transitions within the argyrodite family and further explore how ion hopping is facilitated by lattice dynamics, from long-range phonons to local rotational dynamics of polyanionic species. Through the lens of dynamics spanning multiple time and length scales, we establish guiding principles that govern transport phenomena and highlight avenues of future study for the argyrodite family of ion conductors.

## Graphical abstract

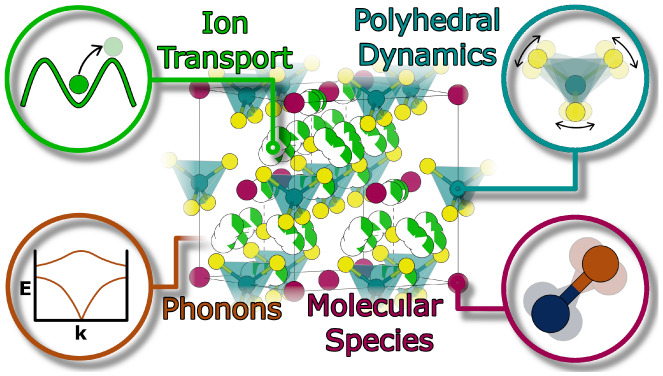

## 1 Introduction

The prototypical argyrodite, Ag_8_GeS_6_ (Figure 1), is a naturally occurring silver ore first identified by Albin Weisbach in 1885.^1^ Ag_8_GeS_6_ served as a feedstock for the first successful isolation of elemental germanium by Clemens Winkler in 1886.^1^ In recent decades, the argyrodite family has expanded to accommodate a wide range of chemistries, which have attracted attention for their optical properties,^2–9^ thermoelectric behavior,^5,10–17^ and their propensity for fast ion conduction.^18–32^ Argyrodites are a compositionally diverse family of chalcogenides with the general form $A_{(12-n-z)/m}^{m+}$ M^n+^ ${\mathrm{Ch}}_{6-z}^{2-}$ X_*z*_ (0 ≤ *z* ≤ 2), where *m* and *n* are the charges of the *A*-cations (mono- or divalent) and *M*-cations (cations from groups 13–15 of the periodic table), respectively.^33^ *Ch* is a chalcogenide, and *X* is a halide or pseudohalide.

**Figure 1:**
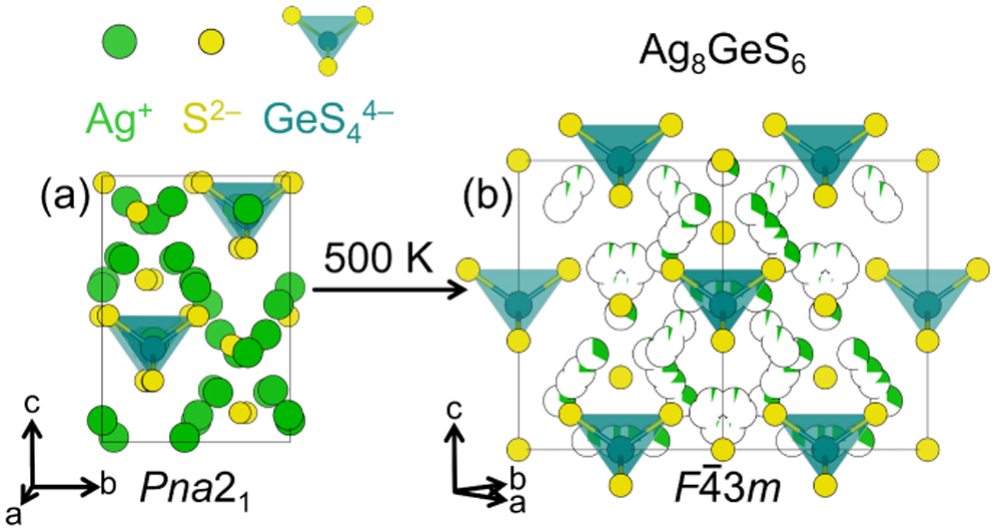
Low temperature (a) and high temperature (b) phases of Ag_8_GeS_6_. Below 500 K, Ag_8_GeS_6_ adopts a *Pna*2_1_ structure with fully occupied Ag^+^ sites. Above 500 K, Ag_8_GeS_6_ adopts the *F*4̅3*m* aristotypic argyrodite structure with highly mobile and delocalized Ag^+^.

Recent interest has been rekindled by Li_6_PS_5_ *X* (*X* = Cl, Br, I) argyrodites and their high room-temperature ionic conductivities (∼10^–3^ S cm^–1^).^18,19^ Through compositional tuning, these conductivities can be further enhanced into the ∼10^–2^ S cm^–1^ range, which is competitive with liquid electrolytes.^34–36^ Ion transport properties of the argyrodites originate from a confluence of crystal structure, atomic disorder, and complex dynamics, including localized dynamics of polyanionic and molecular species as well as long-range vibrations in the form of phonons.

In this perspective, we integrate multiscale structural and dynamic insights to form a holistic understanding of the argyrodites as ion conductors. We first provide a structural overview of the argyrodite family, with particular emphasis on order–disorder phase transitions. We then discuss Arrhenius-type ion transport and review the prevailing structural picture of lithium-ion conduction in argyrodites. Building on this background, we analyze the influence of phonons on ion transport and highlight the critical role of the Arrhenius prefactor (σ_0_) in ionic conductivity. Finally, we transition from collective, periodic dynamics to a local perspective, presenting rotationally active polyhedra and molecular species as key dynamic motifs that govern ion transport.

## 2 The Argyrodite Structure and Its Derivatives

The uniting feature of the argyrodites is the adoption of a high temperature *F*4̅3*m* crystal structure with a disordered and highly mobile *A*-cation sublattice (Figure 1b).^33^ Broadly, the *F*4̅3*m* argyrodite structure is composed of isolated *MCh*_4_ tetrahedra separated by “free” *Ch* ^*2–*^ and *X* ^–^ (where compositionally appropriate). *A*-cations fill the interstices and commonly exhibit partial occupancies across multiple sites. The high temperature *F*4̅3*m* structure is considered an “aristotype” of the argyrodite family—a high-symmetry structure which represents the idealized version of lower-symmetry “hettotypes.”^37^ At low temperatures, various lower symmetry structures are observed (Figure 2a–c), depending on composition.^33,37^ This hettotype-aristotype relationship is illustrated by comparison of the anion sublattices in three common low-temperature structures (Figure 2a–c) with that of the high temperature *F*4̅3*m* aristotype (Figure 2d). In all three hettotypes, the tetrahedral units exhibit slight orientational deviations from their positions in *F*4̅3*m*, while the free chalcogenide sites show complementary modulations. The reduced mobility of cations in the low temperature hettotypes is reflected in localized cation arrangements (Figure 1a) and lower average symmetry.^33^ Table 1 summarizes a selection of Ag^+^, Cu^+^, and Li^+^ argyrodites, their room temperature structures, and their transition temperatures to the *F*4̅3*m* aristotype.

**Figure 2:**
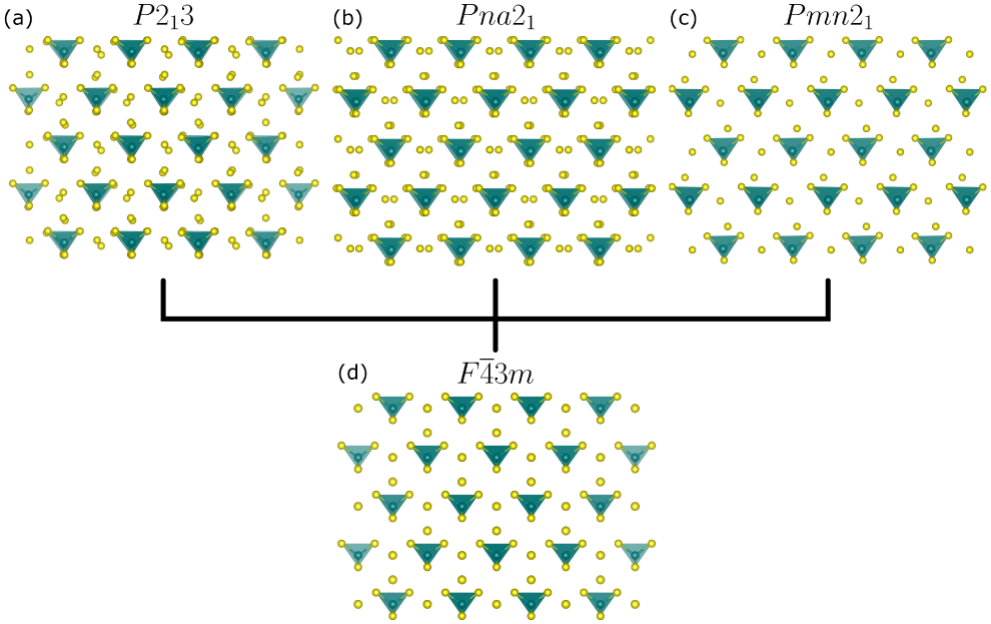
Anion sublattices of (a) *P*2_1_3 (Ag_7_PS_6_), (b) *Pna*2_1_ (Ag_8_GeS_6_), (c) *Pmn*2_1_ (Ag_8_GeSe_6_), and (d) *F*4̅3*m* (Li_6_PS_5_Br) argyrodites. Structures have been expanded and oriented to provide equivalent perspectives that emphasize the translational and rotational modulations that relate the hettotype lattices to the aristotype lattice. Cation sublattices have been omitted for clarity, but do differ between structures. In (d), the mixed S^2–^/Br^–^ sites are colored uniformly yellow for ease of comparison to the hettotype structures.

**Table 1:** Ag^+^, Cu^+^, and Li^+^ Argyrodites with Their Room Temperature Space Groups and Hettotype-Aristotype Transition Temperatures^a^

Composition	Space Group at 298 K	*F*4̅3*m* Transition Temp (K)	Refs
Ag^+^			
Ag_9_GaS_6_	Body-centered orthorhombic	301–310	^55–58^
Ag_8_GaS_5_Cl	*F*4̅3*m*	—	^33^
Ag_8_GaS_5_Br	*F*4̅3*m*	—	^33^
Ag_8_GaS_5_I	*F*4̅3*m*	—	^33^
Ag_9_GaSe_6_	*F*4̅3*m*	281	^59^
Ag_8_GaSe_5_Br	*F*4̅3*m*	—	^33^
Ag_8_GaSe_5_I	*F*4̅3*m*	—	^33^
Ag_8_SiS_6_	*Pna*2_1_	507	^60^,^61^
Ag_7_SiS_5_Cl	*Pna*2_1_	491	^33^
Ag_7_SiS_5_Br	*F*4̅3*m*	—	^33^,^44^
Ag_7_SiS_5_I	*F*4̅3*m*	—	^2^,^33^,^44^,^62^
Ag_8_SiSe_6_	*P*2_1_3 ^14^ *Pmn*2_1_ ^10,11^	370 ^14^ 400 ^10,11^	^10^,^11^,^14^,^61^
Ag_8_SiTe_6_	*F*4̅3*m*	263	^61^,^63^,^64^
Ag_8_GeS_6_	*Pna*2_1_	500	^1^,^61^,^65^,^66^
Ag_7_GeS_5_Cl	*Pna*2_1_	423	^33^
Ag_7_GeS_5_Br	*F*4̅3*m*	—	^33^,^44^
Ag_7_GeS_5_I	*F*4̅3*m*	—	^33^,^44^,^67^
Ag_8_GeSe_6_	*Pmn*2_1_	320–410 ^15^ 321 ^61^	^15^,^61^,^68^
Ag_7_GeSe_5_Br	*F*4̅3*m*	—	^33^
Ag_7_GeSe_5_I	*F*4̅3*m*	—	^31^,^33^,^69^,^70^
Ag_8_GeTe_6_	*F*4̅3*m*	244	^33^,^61^,^71^
Ag_7_GeTe_5_I	*F*4̅3*m*	—	^33^
Ag_8_SnS_6_	*Pna*2_1_	460	^72–74^
Ag_7_SnS_5_Br	*F*4̅3*m*	—	^33^,^44^
Ag_7_SnS_5_I	*F*4̅3*m*	—	^33^,^44^
Ag_8_SnSe_6_	*Pmn*2_1_	355	^29^,^68^,^74^
Ag_7_SnSe_5_I	*F*4̅3*m*	—	^33^
Ag_7_PS_6_	*P*2_1_3	495,^33^ 544 ^66^	^3^,^33^,^66^
Ag_6_PS_5_Cl	*F*4̅3*m*	—	^9^
Ag_6_PS_5_Br	*F*4̅3*m*	—	^9^,^33^,^75^
Ag_6_PS_5_I	*F*4̅3*m*	—	^75^
Ag_7_PSe_6_	*P*2_1_3	453	^76–78^
Ag_6_PSe_5_Cl	*P*2_1_3	—	^79^
Ag_6_PSe_5_Br	*P*2_1_3	—	^33^,^79^
Ag_6_PSe_5_I	*P*2_1_3	—	^33^,^79^
Ag_7_AsS_6_	*P*2_1_3	523	^33^,^80^,^81^
Ag_6_AsS_5_I	*F*4̅3*m*	—	^33^
Ag_7_AsSe_6_	*P*2_1_3	423	^80^
Cu^+^			
Cu_8_SiS_6_	*Pmn*2_1_	336	^17^,^33^,^60^,^74^,^82–85^
Cu_7_SiS_5_I	*F*4̅3*m*	—	^33^,^86^
Cu_8_SiSe_6_	*Pmn*2_1_	323–355	^33^,^74^,^84^,^85^,^87^
Cu_7_SiSe_5_I	*F*4̅3*m*	—	^33^
Cu_8_GeS_6_	*Pmn*2_1_	328–341	^13^,^33^,^74^,^88–90^
Cu_7_GeS_5_I	*F*4̅3*m*	—	^7^,^33^,^86^
Cu_8_GeSe_6_	*P*6_3_ *cm*	983	^84^,^89^,^91^
Cu_7_GeSe_5_I	*F*4̅3*m*	—	^7^,^92^
Cu_7_PS_6_	*P*2_1_3	510	^12^,^33^,^93^
Cu_6_PS_5_Cl	*F*4̅3*m*	241	^33^,^45^,^94^,^95^
Cu_6_PS_5_Br	*F*4̅3*m*	268	^33^,^45^,^94^,^96^
Cu_6_PS_5_I	*F*4̅3*m*	268	^45^,^94^,^96^
Cu_7_PSe_6_	*P*2_1_3	320	^33^,^43^,^97^
Cu_6_PSe_5_Br	*F*4̅3*m*	259	^4^,^16^,^75^
Cu_6_PSe_5_I	*F*4̅3*m*	260–268	^6^,^16^,^75^
Cu_6_AsS_5_Br	*F*4̅3*m*	—	^5^,^98^
Cu_6_AsS_5_I	*F*4̅3*m*	260–280	^5^,^8^,^98^
Li^+^			
Li_7_GeS_5_Br	*F*4̅3*m*	—	^99^
Li_6_PO_5_Cl	*F*4̅3*m*	—	^100^
Li_6_PO_5_Br	*F*4̅3*m*	—	^100^
Li_7_PS_6_	*Pna*2_1_	483	^32^,^46^,^101^,^102^
Li_6_PS_5_Cl	*F*4̅3*m*	—	^18^,^19^,^32^,^46^,^103^,^104^
Li_6_PS_5_Br	*F*4̅3*m*	—	^18^,^19^,^32^,^46^,^103^,^104^
Li_6_PS_5_I	*F*4̅3*m*	173	^18^,^19^,^32^,^46^,^103^,^104^
Li_6_PS_5_BH_4_	*F*4̅3*m*	—	^105^,^106^
Li_6_PS_5_CN	*F*4̅3*m*	—	^107^,^108^
Li_7_PSe_6_	*Pna*2_1_	437	^46^,^101^
Li_6_PSe_5_Cl	*F*4̅3*m*	—	^109^
Li_6_PSe_5_Br	*F*4̅3*m*	—	^109^
Li_6_PSe_5_I	*F*4̅3*m*	—	^110^
Li_6_AsS_5_Br	*F*4̅3*m*	—	^46^
Li_6_AsS_5_I	*F*4̅3*m*	173	^46^
Li_6_AsSe_5_I	*F*4̅3*m*	—	^46^
Li_6_SbS_5_I	*F*4̅3*m*	—	^34^,^111^

a Partial substitutions have been accomplished^20,25,34,50–54^ but have been excluded for brevity.

The hettotype-aristotype relationship is best characterized as an order–disorder transition where translational dynamics of the cations and rotational dynamics of the *MCh*_4_ units are activated. The high symmetry of the *F*4̅3*m* aristotype has long been attributed to dynamic averaging of the *A*-cation sublattice.^33^ We hypothesize that this effect is also at play with the polyhedral anions. Our hypothesis is supported by recent local structure studies that demonstrate local symmetry breaking indicative of *MCh*_4_ rotational dynamics or disorder in aristotypic Li^+^ and Ag^+^ argyrodites.^38–40^ The same local-average structural deviations are not present in low-temperature hettotypes, indicating that disorder is activated upon transition to the aristotype.^39^ Under this formulation, large librations of *MCh*_4_ tetrahedra in the aristotype average to a coaligned and ordered motif (Figures 1b and 2d), while cation hopping is captured as partial occupancy over a wide range of sites (Figure 1b). Similar local-average structural deviations have been rationalized in β-Na_3_PS_4_ and Na_2.9_Sb_0.9_W_0.1_S_4_ as the effect of an order–disorder transition in which the cubic disordered phase dynamically samples various local tetragonal configurations, which average to long-range cubic symmetry.^41,42^

The local structure of Cu^+^-argyrodites is still emerging. We are aware of one study in which temperature-dependent total scattering of Cu_7_PSe_6_ was collected on a laboratory diffractometer with limited resolution.^43^ Notably, the pair distribution function (PDF) exhibited minimal change across the hettotype-aristotype phase transition. These changes were dominated by broadening of pair correlations that may be due to increased thermal displacement or activation of polyhedral dynamics.

The *A*-cation disorder associated with aristotypic argyrodites identified them as candidate ion conductors and prompted the search for variants that adopt the *F*4̅3*m* structure at room temperature.^33^ Although introducing more polarizable chalcogenides often reduces the phase transition temperature (Table 1), such an approach does not reliably stabilize the aristotype at room temperature. In contrast, substitution of the chalcogenide with a halide (or pseudohalide) stabilizes the *F*4̅3*m* aristotype at room temperature across most compositions (Table 1).^33,44–46^ Anion choice is strongly correlated with the amplitude of observed ${\mathrm{PS}}_{4}^{3-}$ rotational disorder in Li_6_PS_5_ *X* (*X* = Cl^–^, Br^–^, I^–^), which implies a link between *MCh*_4_ disorder, anion identity, and phase behavior.^38^ A similar effect is present in Na_3_PO_4_ and Na_3_SbS_4_ derivatives, where introduction of lower valence anions and the resulting cation vacancies stabilize high temperature phases characterized by local polyhedral orientational disorder.^41,42,47,48^ The above observations suggest that weakening anion-anion interactions—through substitution of more polarizable chalcogenides or introducing monovalent halides—stabilizes the argyrodite aristotype by lowering the thermal energy required to activate dynamic disorder, which manifests in local-average structural disagreements.^38–40,49^

## 3 A Brief Primer on Solid-State Ion Transport

In the crystalline solid state, ionic transport is commonly quantified by the Arrhenius framework, in which mobile ions hop between neighboring sites within the periodic structure along a minimum free energy pathway.^112–117^ Between neighboring energetic minima, the ion must pass through an energetic saddle point corresponding to a geometric bottleneck between sites. This construction is rooted in absolute rate theory^118^ and hopping conductivity is described by the Arrhenius relationship:

1 $$\sigma T=\sigma_{0}\exp\left(-\frac{E_{A}}{k_{B}T}\right)$$

where σ is ionic conductivity, *T* is temperature, and *k*_*B*_ is the Boltzmann constant. The activation barrier (*E*_*A*_) includes the enthalpy of migration (Δ*H*_*m*_) and defect formation energy $\left(\frac{1}{2}\Delta H_{f}\right)$ , while the Arrhenius prefactor (σ_0_) represents temperature-independent contributions to ion transport:

2 $$\sigma_{0}=\left(\frac{\gamma c\nu_{0}Z^{2}e^{2}a_{0}^{2}}{k_{B}}\right)\exp\left(\frac{\Delta S_{m}}{k_{B}}\right)$$

σ_0_ contains contributions from the dimensionality of ion conduction pathways (γ), number and charge of mobile carriers (*c*, *Z*), the elementary charge (*e*), the attempt frequency of ion hopping (ν_0_), and the jump distance (*a*_0_).^117,119,120^ σ_0_ is exponentially dependent upon entropy of migration (Δ*S*_*m*_), which is determined (under the harmonic approximation) by the ratio of the vibrational partition functions associated with the mobile ion:^118^

3 $$\Delta S_{m}=k_{B}\ln\left(\frac{\overset{N-1}{\underset{j=1}{\prod }}\nu_{j}^{0}}{\overset{N-1}{\underset{j=1}{\prod }}\nu_{j}^{\prime }}\right)$$

where the numerator is a product over *N* – 1 normal modes of the system with the mobile ion in the equilibrium position and the denominator is a product over *N* – 1 normal modes when the mobile ion is at the saddle point.^118^

The above relationships decompose temperature-dependent ionic conductivity into basic energetic terms, but imply discrete and entirely uncorrelated hopping processes. This framework does not necessarily hold for solid ion conductors in which ion motion is often highly correlated. This phenomenon is described by the Haven ratio, *H*_*R*_:

4 $$H_{R}=\frac{D^{*}}{D_{\sigma }}$$

where D* is the tracer or self-diffusivity of a mobile ion and *D*_σ_ is the charge or conduction diffusivity calculated from ionic conductivity.^53,121–123^ Self-diffusivity describes the ease with which a single mobile ion moves through the electrolyte, while the conduction diffusivity describes the macroscopic propensity for a material to move charge. In systems where the mobile species are either extremely dilute or completely noninteracting, D* = *D*_σ_ and *H*_*R*_ = 1.^121^ *H*_*R*_ < 1 reflects positively correlated motion that enhances net charge transport, while *H*_*R*_ > 1 indicates negatively correlated or closed-loop motion that leads to low charge transport relative to self-diffusion.^122^ Ceramic fast ion conductors often have *H*_*R*_ values of less than 1 and exhibit correlated motion that enhances charge transport.^53,121–123^ While *H*_*R*_ indicates the presence of correlated motion, it provides minimal insight into the mechanistic character of correlation effects.^123^ Li_6_PS_5_Cl is characterized by *H*_*R*_ < 1,^53,124^ while a comparison of ionic conductivities estimated from nuclear magnetic resonance (NMR) spectroscopy and those determined via broadband impedance spectroscopy indicates that *H*_*R*_ ≈ 1 in Li_6_PS_5_Br and *H*_*R*_ ≫ 1 in Li_6_PS_5_I.^125^

Absolute rate theory assumes statistically independent hopping events, yet the deviation of the Haven ratio from unity may suggest that the Arrhenius framework is not suitable for describing correlated hopping events. In some systems, often “superionic” conductors, absolute rate theory breaks down and a “liquid-like” diffusive regime emerges.^126^ The failure of absolute rate theory is indicated by a multiple orders of magnitude deviation of the rate prefactor obtained via NMR from the optical phonon frequencies of the crystal.^127,128^ This behavior is not observed in the Li_6_PS_5_ *X* (*X* = Cl^–^, Br^–^, I^–^) argyrodites that are central to this work—ν_0_ obtained from ^7^Li NMR is within 1 order of magnitude of the Debye frequency (ν_*D*_ ≃ 10^12^ Hz), which provides an indicator of phonon frequency, for all three compositions.^18,49^ In these systems, ion transport can be treated under absolute rate theory despite deviations of *H*_*R*_ from unity because diffusion proceeds via chain-like hopping rather than uncorrelated nearest-neighbor hops.^22,129^ In anion-disordered phases (Section 4), long, open diffusion chains allow Δ*H*_*f*_ to be interpreted as the energy required to form Frenkel pairs separated by a chain length rather than adjacent defects.^129^ In anion-ordered phases, closed diffusion chains within Li^+^ cages (Section 4) produce high local mobility and low macroscopic conductivity, with Arrhenius behavior reflecting the rate-limiting transfer between cages.^22,125^

## 4 The Structural Picture of Ion Transport in Argyrodites

Although this perspective primarily examines the dynamics of the argyrodites, it is important to first outline the structural basis of ion transport. Here we discuss how structure dictates ion transport in Li_6_PS_5_ *X* (*X* = Cl^–^, Br^–^, I^–^) and their derivatives. We focus our discussion on aristotypic Li^+^-argyrodites due to the ubiquity of high-quality structural and electrochemical studies in these systems.^18–25,27,28,34,35,53,130^ We highlight possible pathways for Li^+^ hopping processes and establish the structure–property relationships that facilitate fast ion transport.

Fast ion conduction in argyrodites arises from formation of a percolating network of tetrahedral voids, commonly described by doublet, intracage, and intercage hops. The Li^+^ sublattice consists of pseudo-octahedral cages centered at the 4*d* Wyckoff position (Figure 3a and b), whose vertices can host Li^+^ and support rapid migration between clustered sites (doublet, Figure 3c).^21^ While ion motion within cages is fast (≈10^9^ s^–1^ at 329 K in Li_6_PS_5_I)^131^ long-range transport is limited by intercage hops between neighboring cages. Understanding ion transport therefore requires resolving the possible physical pathways that collectively define these processes.

**Figure 3:**
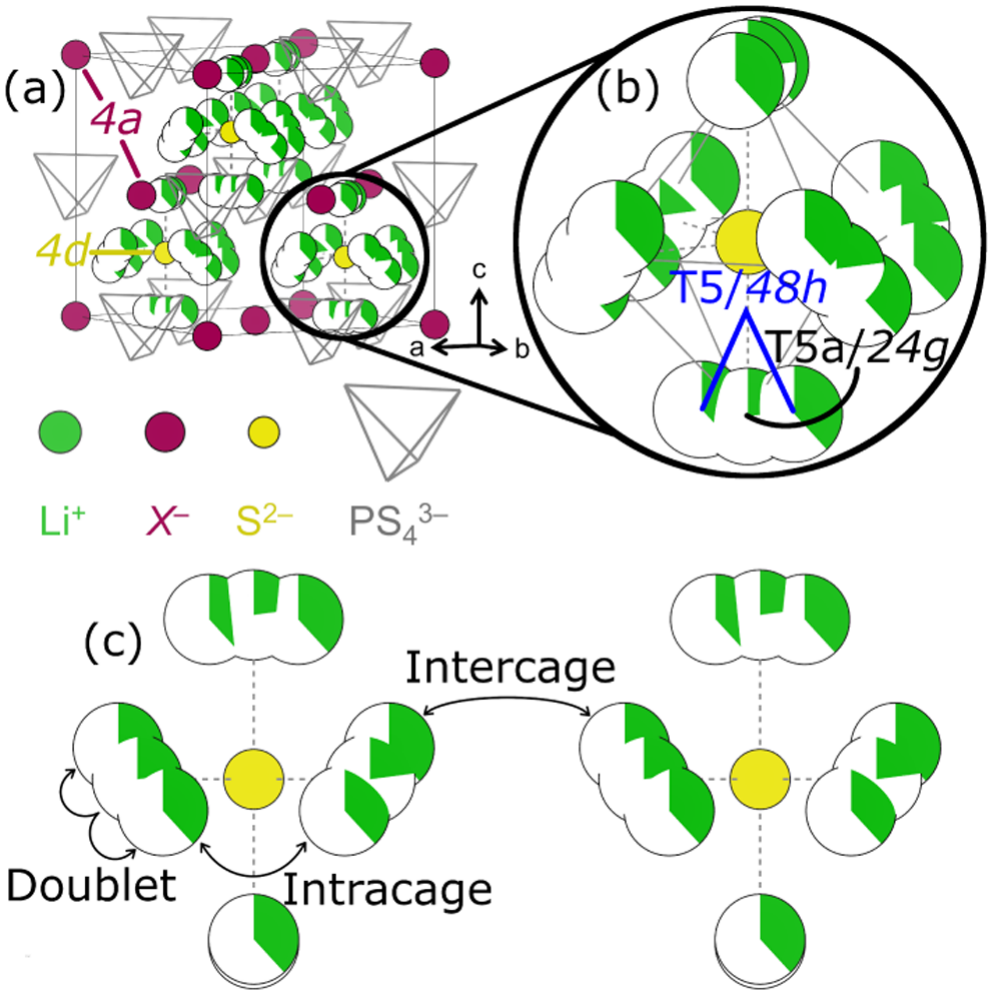
Illustration of (a) the structure of a Li_6_PS_5_ *X* argyrodite exhibiting no S^2–^/*X* ^–^ antisite disorder. *X* ^–^ occupies the 4*a* Wyckoff position on the faces and corners of the unit cell, while S^2–^ occupies the 4*d* positions on the interior of the unit cell. Li^+^ is arranged in pseudo-octahedral cages on the 48*h* and 24*g* Wyckoff positions surrounding the 4*d* site (b). Cations can hop within and between cages (c). All three illustrated hop types are required for long-range ion transport.

Five tetrahedral sites (types 1–5) can accommodate Li^+^ in argyrodites, with Li^+^ most commonly observed on type 5 (T5, Figure 4a), type 2 (T2, Figure 4b), and type 4 (T4, Figure 4c) sites.^19,24,25,35,46,132^ Significant Li^+^ density is also found at the shared face of adjacent T5 sites (T5a), though it remains unclear whether this represents a true equilibrium site or time-averaged hopping between T5 positions.^19,132^ T5 sites are coordinated by 4*a*, 4*d*, and two S^2–^ anions from distinct ${\mathrm{PS}}_{4}^{3-}$ units; T2 sites share a similar coordination but draw both S^2–^ from the same ${\mathrm{PS}}_{4}^{3-}$ unit, while T4 sites are coordinated by 4*a* and three S^2–^ from different ${\mathrm{PS}}_{4}^{3-}$ units. Minor occupation of T3 sites, which share faces only with T4, has been reported and may indicate a T4-T3-T4 pathway.^25,133^ Within this framework, T5/T5a sites are treated as equilibrium positions, while T2 and T4 act as interstitial sites. Doublet hopping occurs directly between adjacent T5 sites, intracage hopping proceeds via T5-T2-T5, and intercage transport follows either T5-T2-T2-T5 (Figure 4d) or T5-T4-T5 (Figure 4e) pathways.^22^

**Figure 4:**
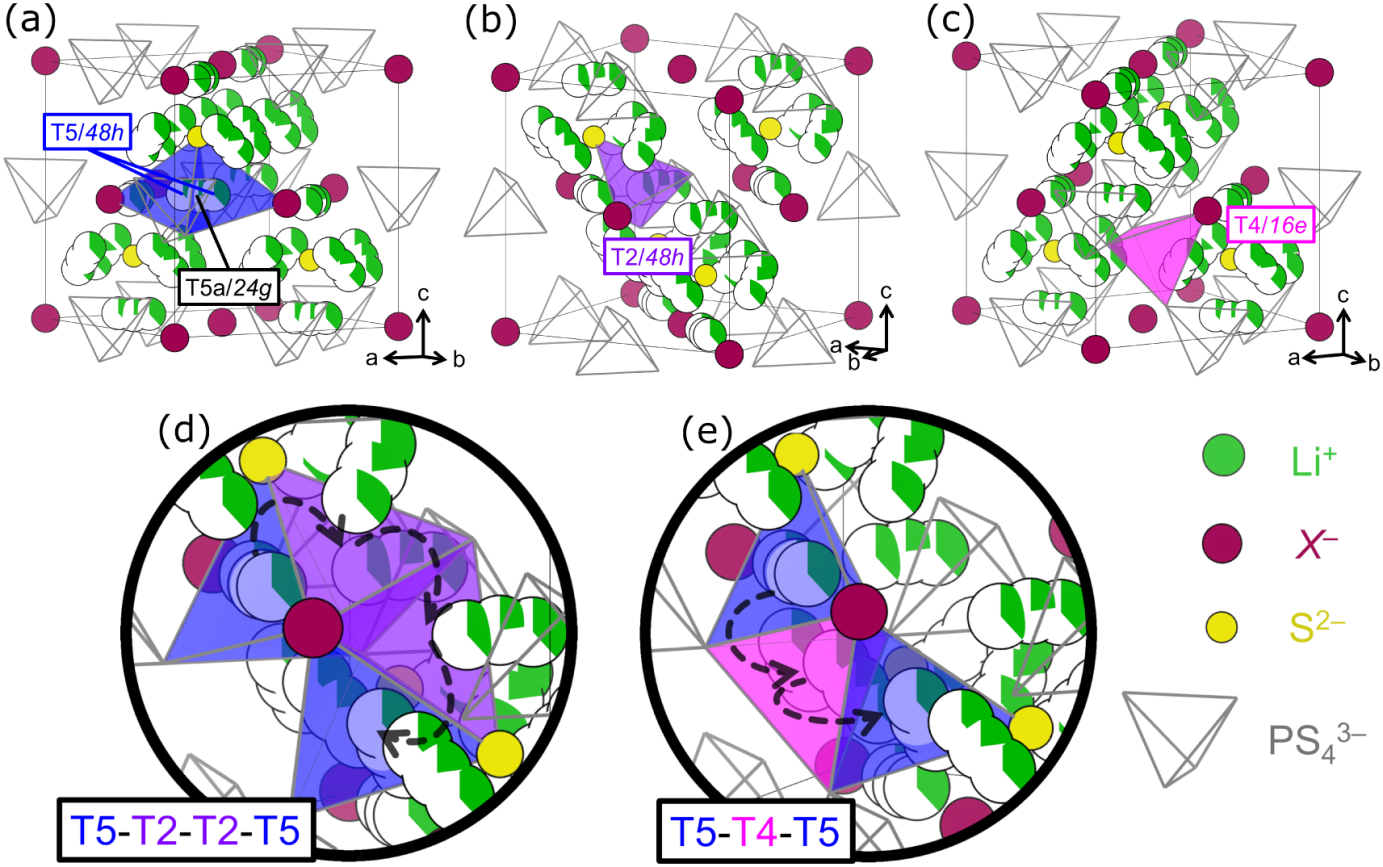
Tetrahedral voids and corresponding Li^+^ sites that have been reported with significant occupation in the Li_6_PS_5_ *X* (*X* = Cl^–^, Br^–^, I^–^) system and its derivatives. The T5 and T5a sites (a) exhibit the highest occupancy and represent equilibrium positions. Population of the T2 (b) and T4 (c) sites is associated with fast ion conduction due to activation of T5-T2-T2-T5 (d) and T5-T4-T5 (e) intercage hopping pathways.

Anion antisite disorder is a well-established materials design principle for high ionic conductivity in Li^+^ argyrodites. Li_6_PS_5_I represents the anion-ordered case; I^–^ almost exclusively occupies the 4*a* Wyckoff position, while S^2–^ occupies the 4*d* Wyckoff position (Figure 3a), which has been attributed to the large ionic radius of I^–^.^18^ In contrast, Li_6_PS_5_Cl and Li_6_PS_5_Br exhibit S^2–^/*X* ^–^ antisite disorder— the 4*a* and 4*d* sites exhibit partial occupation of both S^2–^ and *X* ^–^.^18^ The presence of anion site mixing in Li_6_PS_5_Cl and Li_6_PS_5_Br leads to ionic conductivities 3 orders of magnitude greater than Li_6_PS_5_I.^18,134^ This increase in ionic conductivity arises from activation of the intercage T5-T2-T2-T5 and T5-T4-T5 hops needed for long-range ion conduction.^22^ In anion-ordered systems, intercage pathways have large energy barriers. Mobile ions are confined to motions within the cages, resulting in low bulk ionic conductivity.^21,22,134–136^ Figure 5 illustrates this behavior in Li_6_PS_5_Br.^136^

**Figure 5:**
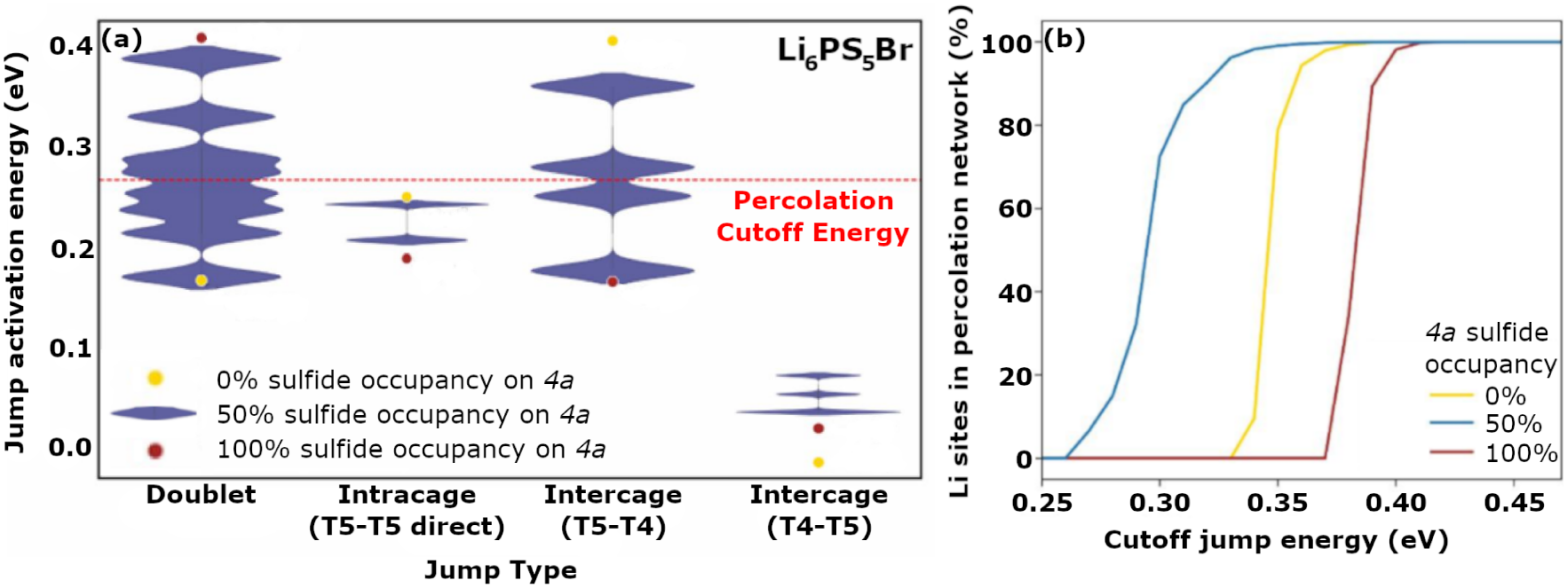
Local anion occupation on the 4*a* and 4*d* Wyckoff positions significantly influences the activation energies of doublet, intracage, and intercage transport pathways in Li_6_PS_5_Br (a). When anion site disorder is introduced, a variety of local environments are present (blue violin plots, width represents the relative prevalence of each local jump environment at 50% sulfide occupancy on 4*a*), which allow the formation of a percolating network at a lower cutoff energy than in either ordered configuration (b). Intracage hopping was treated as direct T5-T5 hopping, resulting in higher activation energies than those calculated for the T5-T2-T5 intracage pathway.^135^ Activation energies associated with the T5-T2-T2-T5 follow the same trend as those for the T5-T4 intercage hops as a function of site disorder.^135^ Adapted from ref 136. Copyright 2025 American Chemical Society.

Anion antisite disorder enables the formation of percolating ion transport pathways by averaging the electrostatic charge of the anions and broadening the distribution of local electrostatic environments for Li^+^. When S^2–^ exclusively resides on the 4*d* site (as in Li_6_PS_5_I), Li^+^ remains localized in cages around the S^2–^ on the 4*d* site.^22^ This favors the T5-T5 doublet hop, but strongly disfavors the T5-T4-T5 and T5-T2-T2-T5 hops needed for intercage transport (Figure 5a). When S^2–^ exclusively occupies the 4*a* site, the Li^+^ cages form around that site, allowing low-energy T5-T2-T2-T5 and T5-T4-T5 hopping, but raising the activation barrier for the T5-T5 doublet hop. Introducing site disorder distorts the octahedral Li^+^ cages and generates an ensemble of local environments with varying S^2–^ and *X* ^–^ arrangements across 4*a* and 4*d* sites, which change the activation energies of nearby hopping pathways.^22^ With sufficient site disorder, local environments that favor doublet hopping will have neighboring environments that favor intercage hopping (intracage hops are always accessible), allowing Li^+^ to percolate through locally favorable environments with lowered activation barriers (Figure 5b).^22,135,136^ We note that a broader distribution of electrostatic environments is more important for the formation of low-energy percolating pathways than simply lowering the charge density of the anion at the center of the Li^+^ cages. This design principle is further supported by compositional derivatives of the argyrodite family. In multianion argyrodites such as Li_5.5_PS_4.5_Cl_*x*_Br_1.5–*x*_ ^137^ and Li_6_PS_5_(CN)_1–*x*_Br_*x*_,^138^ the additional disorder introduced by distributing all three anions across both the 4*a* and 4*d* sites results in lower average activation barriers and higher ionic conductivities relative to the single-(pseudo)­halide analogs. Similarly, halide-enrichment in the Li_6–*y*_PS_5–*y*_ *X*_1+*y*_ (*X* = Cl^–^, Br^–^) argyrodites reduces the total average charge distributed across the 4*a*/4*d* sites and enforces anion site disorder, lowering the average activation barriers of the three hop types and converging them toward the same value.^27,28,35,53,135,136^

While it is convenient to discuss ion transport in the argyrodites as occurring via discrete hops along specific pathways, Figure 5 shows that anion disorder results in a broad distribution of energies associated with these pathways. Yet, temperature dependent ionic conductivity measurements yield single values for activation energy and Arrhenius prefactor. This seeming contradiction suggests that analysis of discrete hops, while conceptually useful, belies a regime characterized by highly correlated ion motion. The Haven ratio of 0.456 for Li_6_PS_5_Cl supports this notion.^124^

## 5 Phonons

The dynamics of ion motion in solid-state ion conductors—particularly the coupling between lattice vibrations and mobile ions—are increasingly recognized as central to fast ion conduction. It is useful to consider these motions in terms of phonons: collective and coherent excitations of the lattice.^139,140^ Reducing phonon frequencies results in an increased phonon population at a given temperature, and elevated phonon populations enable larger ion displacement amplitudes that facilitate ion hopping.^117,120,141,142^ In this section, we examine the role of phonons in phase transitions and ion transport in the argyrodites.

### 5.1 Phase Transitions and Phonon “Melting”

The high-temperature aristotype of the argyrodites has long been understood to be a consequence of heavy disordering in the cation sublattice, which generates a high symmetry average structure.^33^ In halide-free Ag^+^ and Cu^+^ argyrodites (e.g., Cu_7_PSe_6_ ^43,97^ Cu_7_PS_6_ ^93^ Ag_8_GeSe_6_ ^143^ Ag_8_SnSe_6_,^29^ the phase transition can be classified as an order–disorder transition in which the mobile ion sublattice “melts” while the anion sublattice remains relatively rigid. This “melting” is accompanied by a collapse of cation phonon modes into a heavily overdamped and anharmonic regime where these phonon frequencies approach zero energy and the oscillatory behavior of the system is suppressed. The breakdown of these phonons coincides with the onset of a dynamic regime in which the mobile ion sublattice exhibits liquid-like “superionic” transport.^29,43,93,97,139,143,144^ In contrast, the anion sublattice remains largely ordered on average, with only minor modulations of the polyhedral units and free chalcogenides in the time-averaged structure (Figure 2).^93^ Anharmonicity and phonon softening enable many argyrodites to exhibit ultralow lattice thermal conductivity relevant for applications in thermoelectrics.^29,43,97,145^

Cation “melting” is illustrated by the Ag_8_SnSe_6_ system, which exhibits a sharp phase transition from the low temperature *Pmn*2_1_ structure to the high temperature *F*4̅3*m* structure at *T* = 355 K.^29^ Concomitant with this phase transition, the inelastic neutron spectrum (INS) evolves from quasi-harmonic to quasi-elastic, overdamped behavior with increasing temperature (Figure 6a).^29^ This evolution is consistent with Ag^+^ ions switching from oscillating about their equilibrium positions (quasi-harmonic) to rapid diffusion (quasi-elastic). The cation sublattice “melting” is captured in structural data through significant disordering of Ag^+^ paired with only minor reordering of the anion sublattice (Figure 6b and c).

**Figure 6:**
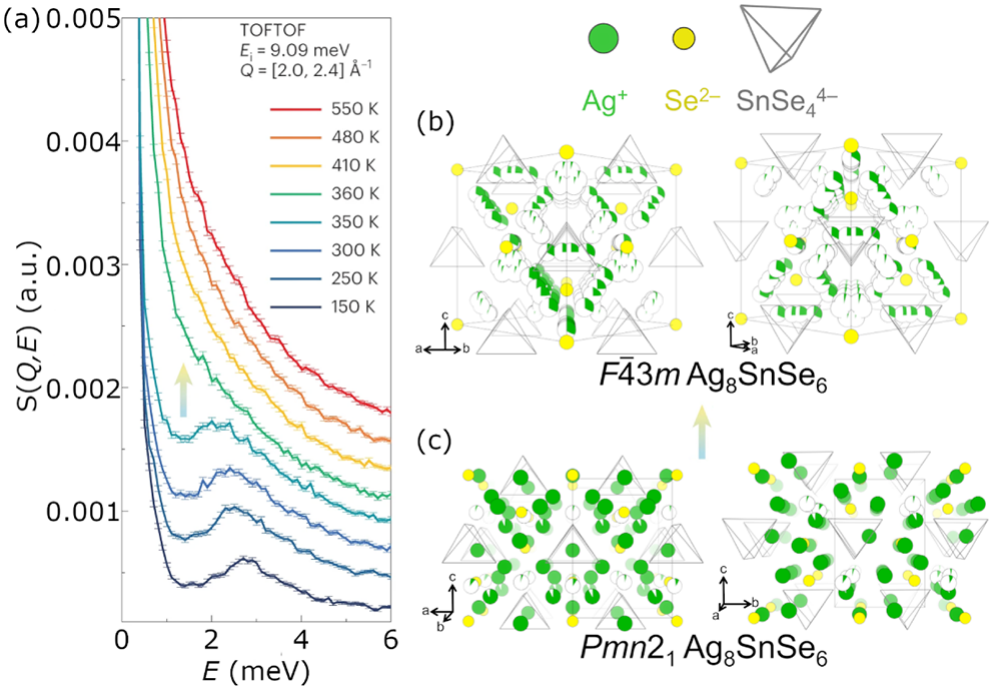
Ag_8_SnSe_6_ exhibits “melting” of Ag^+^ sublattice reminiscent of a first order phase transition. This is characterized by the onset of a quasi-elastic component in the inelastic neutron spectrum (INS, integrated over a *Q* range of 2.0–2.4 Å^–1^) between *T* = 350–360 K that is coincident with the phase transition at 355 K (a). From a structural perspective, this “melting” behavior can be seen in the change from an ordered Ag^+^ sublattice in the low temperature *Pmn*2_1_ structure (c) to a disordered Ag^+^ sublattice in the high temperature *F*4̅3*m* structure (b). Panel (a) adapted with permission from ref 29. Copyright 2023 Springer Nature.

The above representation becomes more complex when multiple phase transitions are present over a narrow temperature range, as is the case for Ag_8_GeSe_6_ and Cu_7_PSe_6_.^43,97,143^ Stepwise “melting” of the cation sublattice appears to occur, where each phase transition further disorders the cation sublattice. One possible explanation of this behavior is that the first hettotype-hettotype transition activates intracage ion diffusion (discussed in Section 4), while the hettotype-aristotype transition activates intercage ion diffusion and thereby allows long-range ion transport. Cu_7_PSe_6_ exhibits a transition from the low temperature *Pna*2_1_ phase to the intermediate temperature *P*2_1_3 phase at *T* = 250 K.^97^ Concomitant with this phase transition, localized oscillations of the Cu^+^ sublattice give way to facile intracage ion transport and a quasi-elastic behavior appears in the INS (Figure 7a–c).^97^ At *T* = 320 K, the *F*4̅3*m* structure is adopted and intercage transport is activated, at which point the cation melting transition is fully completed (Figure 7a,c,d).^97^ The presence of multiple phase transitions in Cu_7_PSe_6_ has been attributed to the lower stability of *d* ^10^ Cu^+^ in low coordination number environments with Se^2–^, which localizes Cu^+^ ions in the low-temperature *Pna*2_1_ structure.^76,146,147^ In systems that undergo a single phase transition, such as Ag_8_SnSe_6_, both types of diffusion are activated simultaneously.

**Figure 7:**
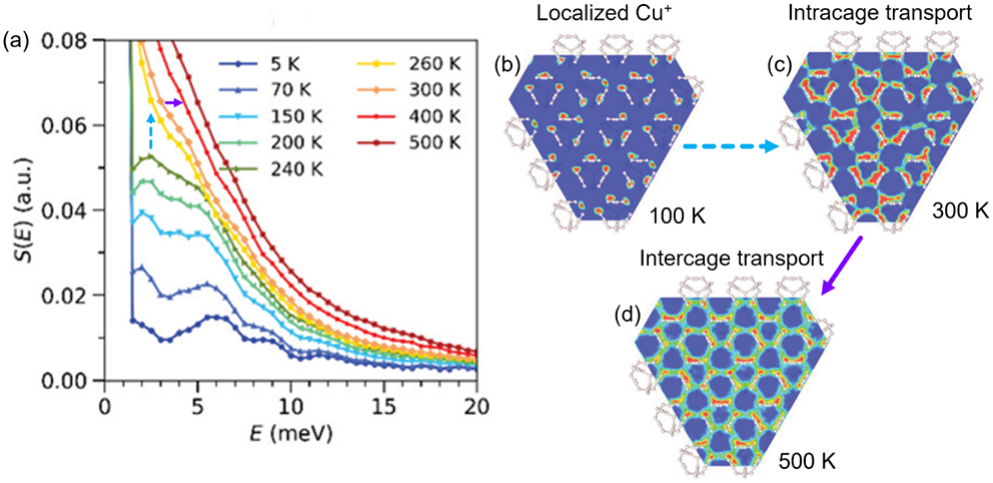
Cu_7_PSe_6_ exhibits phase transitions at 250 K and at 320 K that occur concomitant with cation sublattice “melting”. (a) The inelastic neutron spectrum (INS) integrated over a *Q* range of 1–4 Å^–1^ exhibits the onset of quasi-elastic behavior coincident with the 250 K transition (blue arrow). Cu^+^ probability density maps of the (111) plane in the *F*4̅3*m* structure calculated from machine learned molecular dynamics simulations (b–d) show that this transition induces facile intracage transport (c). The phase transition to the *F*4̅3*m* structure at 320 K (purple arrow) allows for facile intercage hopping at elevated temperatures and completes the “melting” process (d). Adapted with permission from ref 97. Copyright 2022 John Wiley and Sons.

While halide-free Ag^+^ and Cu^+^ argyrodites both appear to exhibit “superionic” transitions characterized by cation sublattice melting, the halide argyrodites (e.g., *A*_6_ *MCh*_5_ *X*) appear to exhibit distinct phase transition behavior. Li_6_PS_5_Cl is only known to adopt the *F*4̅3*m* aristotypic structure down to 5 K, as determined by neutron powder diffraction.^148^ Inelastic and quasielastic neutron scattering studies on ^7^Li_6_PS_5_Cl indicate that the mobile ion sublattice exhibits progressive “melting” behavior from 50–600 K.^149^ This behavior runs counter to the sharp cation sublattice “melting” transitions observed in halide-free Ag^+^ and Cu^+^ argyrodites and indicates that halide substitution fundamentally changes the behavior of the cation and polyhedral dynamics that participate in the phase transitions. Recent studies have found that the local structures of the Li_6_PS_5_ *X* argyrodites are not well-described by the *F*4̅3*m* aristotype.^38,40^ Rather, these deviations have been modeled by both supercell modulations and by pseudo-random rotational disorder of the ${\mathrm{PS}}_{4}^{3-}$ tetrahedra. Preservation of the *F*4̅3*m* aristotype to low temperatures combined with the local structure may suggest dynamic averaging of ${\mathrm{PS}}_{4}^{3-}$ dynamics, with higher temperatures resulting in higher-amplitude librations.

The onset of an overdamped, anharmonic regime calls into question the description of discrete hopping behavior discussed in Sections 3 and 4. Can cation transport in aristotypic argyrodites usefully be described as ions hopping between sites, or does absolute rate theory lose its validity? In many systems that are poorly described by absolute rate theory, transition to the superionic phase is accompanied by a discontinuous drop in *E*_*A*_,^127^ as is observed in the Cu^+^ and Ag^+^ superionic argyrodites.^43,145^ In contrast, the lithium halide analogs appear to reside in the hopping regime at or near room temperature. Below *T* = 400 K, Li_6_PS_5_Cl exhibits a quadratic, Debye relationship between the vibrational density of states (VDOS) and phonon frequency (*g*(ω) ∝ ω^2^).^149^ At *T* = 400 K, the INS develops quasi-elastic broadening characteristic of fast hopping or diffusion. At *T* = 600 K, the liquid-like regime is established, as evidenced by the linear relationship between VDOS and phonon frequency (*g*(ω) ∝ ω). For Li_6_PS_5_Cl and the halide congeners, the liquid-like “superionic” regime is therefore not present at or near room temperature. As such, Arrhenius hopping and absolute rate theory are expected to be a valid framework for understanding ion transport at temperatures where ionic conductivities are typically measured experimentally.

### 5.2 Activation Energy (*E_A_*)

The activation barrier for ionic conduction is strongly influenced by the phonons of the host lattice. The lowest energy optical phonon (LEO)—the lowest-frequency phonon where atoms move out of phase with one another—is important for ion transport as it plays a dominant role in displacing the mobile ion relative to the anions.^141^ Early work by Wakamura established a correlation between the LEO phonon frequencies and activation energies across a variety of ion conductors, specifically in materials where mobile ion vibrational amplitudes exceed those of the framework ions.^141,150^ Softening of these LEO modes and the corresponding reduction in activation energy are further linked to changes in the dielectric constants of ion conductors. As polarizability and dielectric constant increase, both activation energy and LEO phonon frequency tend to decrease.^141,151,152^ This phonon-transport coupling arises from lattice softening effects—as the polarizability of constituent anions increases, more phonon modes become thermally accessible at a given temperature, leading to generally lower activation energies.^120,141,150^ The physical basis for this relationship can be rationalized in the context of Pearson hard–soft acid–base theory: softer, more polarizable species can more effectively screen the electrostatic interactions with relatively hard cations, such as Li^+^.^117,153^ Thus, lattice softening creates shallower potential wells, and ions can access a wider portion of those wells at the same energetic cost, increasing the probability that a mobile ion will migrate into a neighboring stable site.

In Li_6_PS_5_ *X* argyrodites, halide and chalcogenide substitution modify *E*_*A*_ for Li-ion transport through competing effects of lattice softening and anion site ordering. As shown in Figure 8, *E*_*A*_ decreases linearly with increasing Br^–^ content in Li_6_PS_5_Cl_1–*x*_Br_*x*_.^18^ Similarly, Se^2–^ substitution in Li_6_PS_5–*x*_Se_*x*_I results in a monotonic decrease in *E*_*A*_ and yields a 2 orders of magnitude improvement in ionic conductivity.^110^ Both of these cases are consistent with the general principle that increasing lattice polarizability lowers *E*_*A*_. This general rule can be weaker than structure effects, specifically anion ordering. Based on polarizability, substitution of Br^–^ with I^–^ would be expected to decrease *E*_*A*_. In practice, introducing I^–^ leads to ordering of S^2–^ and *X* ^–^ anions on the 4*d* and 4*a* sites concurrent with an increase in *E*_*A*_.^18^ Anion ordering also occurs in Li_6_PS_5–*x*_Se_*x*_Br, where addition of Se^2–^ results in a slight increase in both *E*_*A*_ and ionic conductivity.^154^ Full ordering of the anion species increases *E*_*A*_ for the rate limiting intercage hop and generally exerts a stronger influence than anion polarizability, as detailed in Section 4.^18,20–23^ In light of these case studies, both site disorder and anion polarizability exert strong influences on *E*_*A*_ in Li_6_PS_5_ *X* argyrodites.

**Figure 8:**
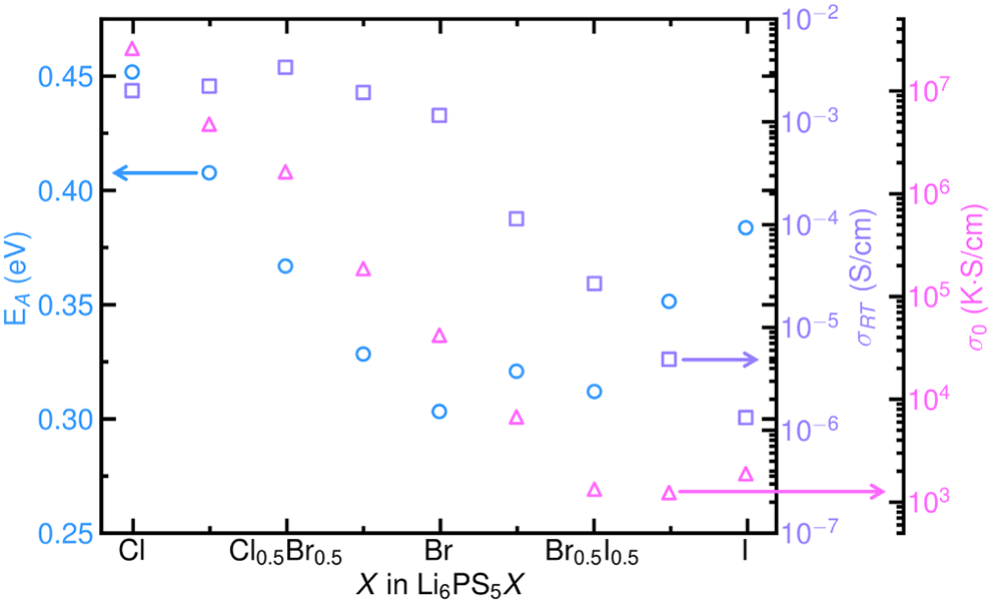
Activation energies (*E_A_*, blue circles), Arrhenius prefactors (σ_0_, pink triangles), and room temperature ionic conductivities (σ*_RT_*, purple squares) for Li_6_PS_5_ *X* (*X* = Cl^–^, Br^–^, I^–^) and alloy compositions. *E_A_* decreases with halide softening as Cl^–^ is replaced with Br^–^, but upon introduction of I^–^, *E_A_* increases due to anion site ordering. σ_0_ decreases with halide softening as Cl^–^ is replaced with Br^–^. Further softening by addition of I^–^ continues to decrease σ_0_ until the Li_6_PS_5_Br_0.5_I_0.5_ composition is reached. Additional I^–^ substitution exhibits a minimal effect on σ_0_. Data reproduced from ref 18.

### 5.3 Arrhenius Prefactor (σ_0_)

Until recently, *E*_*A*_ has dominated discussions of ion conduction, while the prefactor (σ_0_)—which contains terms with significant influence on ionic transport—has often been neglected. As shown in eq 2, σ_0_ is a collection of temperature-independent terms and is determined via temperature-dependent ionic conductivity measurements.

In Li_6_PS_5_ *X* (*X* = Cl^–^, Br^–^, I^–^), several terms in the prefactor remain relatively constant. γ and *Ze* remain unchanged due to fixed pathway dimensionality and ion charge. The lattice parameter varies by only ≈ 3% from Li_6_PS_5_Cl to Li_6_PS_5_I,^18^ so changes in *a*_0_ and *c*—which is not expected to change significantly with constant Li^+^ content—cannot account for the observed 3 orders of magnitude difference in σ_*RT*_. Tuning Li^+^ content through aliovalent substitution of P^5+^ or by varying the S^2–^/*X* ^–^ ratio is expected to impact *c*, but we focus this section on materials with fixed Li^+^ content.^20,25,28,35,53^ For Li_6_PS_5_ *X* (*X* = Cl^–^, Br^–^, I^–^), ν_0_ and Δ*S*_*m*_ are primary drivers for variation in σ_0_ and are intimately connected to dynamics. ν_0_ is the frequency that the mobile ion attempts a hop and is generally accepted to be synonymous with the frequency at which the mobile ion oscillates about its equilibrium position.^119^ If a mobile ion oscillates at a higher frequency and makes more hopping attempts with a fixed probability of success, it will succeed more often and the ionic conductivity will increase.

The Debye frequency (ν_*D*_) is generally accepted to be a reasonable approximation of attempt frequency in absolute rate theory.^112^ ν_*D*_ is the cutoff frequency in the Debye model that approximates the maximum frequency of acoustic phonon modes in the crystal.^155^ The Debye temperature, θ_*D*_, is defined as $\theta_{D}\equiv h\nu_{D}k_{B}^{-1}$ and corresponds to the temperature at which all normal modes are thermally accessible.^155^ Physically, ν_*D*_ corresponds to the frequency at which the phonon wavelength is on the order of the unit cell dimensions and represents the shortest wavelength consistent with the crystal periodicity.^156^ If the speed of sound within a structure is assumed constant for all frequencies, ν_*D*_ can be calculated according to:

5 $$\nu_{D}={\left(\frac{3}{4\pi V}\right)}^{1/3}v$$

where *V* is the volume of the unit cell and *v* is the speed of sound through the material.^18,155,156^

Δ*S*_*m*_ appears to be a stronger driver of the magnitude of the Arrhenius prefactor in argyrodites than ν_*D*_. Speed of sound measurements of the Li_6_PS_5_Cl_1–*x*_Br_*x*_, Li_6_PS_5_Br_1–*x*_I_*x*_, and Li_6_PS_5–*y*_Se_*y*_I compositional series reveal the general trend that the introduction of larger and more polarizable anions reduces the magnitude of the ν_*D*_. For Li_6_PS_5_Cl_1–*x*_Br_*x*_ and Li_6_PS_5–*y*_Se_*y*_I, ν_*D*_ decreases monotonically and mirrors the trends in *E*_*A*_ (Figures 8 and 9a), supporting the case that lattice polarizability lowers E_*A*_.^18,110^ ν_*D*_ alone is insufficient to explain trends in σ_0_; σ_0_ is expected to scale linearly with ν_0_ (approximated by ν_*D*_), but this is not observed in either Li_6_PS_5_ *X* or Li_6_PS_5–*y*_Se_*y*_I. In Li_6_PS_5_ *X*, ν_*D*_ varies by only ∼25% and σ_0_ spans 4 orders of magnitude, indicating that ν_0_ is not the primary driver of σ_0_ values.^18^ Similarly, ν_*D*_ varies by a factor of 2 in Li_6_PS_5–*y*_Se_*y*_I (Figure 9c), while σ_0_ varies by a full order of magnitude.^110^ As such, the linear relationship between ν_0_ and σ_0_ (σ_0_ ∝ ν_0_) is insufficient to explain trends in σ_0_. Instead, σ_0_ appears to be exponentially correlated with halide occupancy on the 4*d* site (σ_0_ ∝ *e* ^*disorder*^, Figure 9b and d). This relationship mirrors the dependence of σ_0_ on Δ*S*_*m*_ $\left(\sigma_{0}\propto e^{\Delta S_{m}}\right)$ and implies that halide occupancy on the 4*d* site generates a broadened distribution of vibrational environments, which increases Δ*S*_*m*_ and drives large changes in σ_0_. Confirmation of this dependence requires calculating or estimating Δ*S*_*m*_.

**Figure 9:**
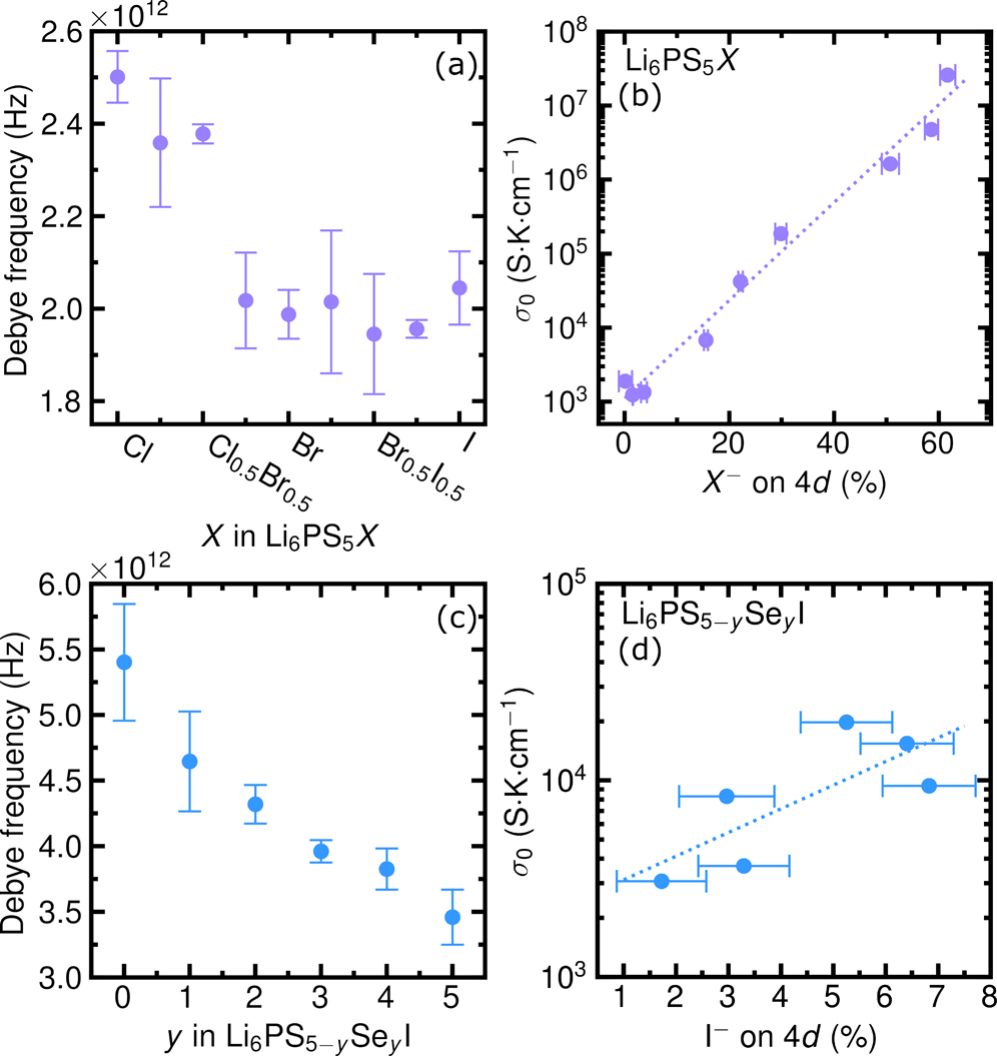
Debye frequencies (ν*_D_*) are reduced with increasing halide (a) and chalcogenide (c) polarizability. Arrhenius prefactors (σ_0_) trend exponentially with halide occupancy on the 4*d* Wyckoff position (b, d). Dotted lines in (b) and (d) are guides to the eye. Data reproduced from ref 18,110 .

A method of calculating Δ*S*_*m*_ is emerging in the solid-state ionics literature. This method relies on reframing the Arrhenius relationship in eq 1 from terms of ionic conductivity to hopping frequency, thereby eliminating most of the linear terms in the prefactor.^27,138,157,158^ This allows for calculation of Δ*S*_*m*_ given hopping frequency, Δ*H*_*m*_, and attempt frequency. Hopping frequency can be obtained from the characteristic frequency of the equivalent circuit element representing bulk ion transport in EIS.^27,157^ Δ*H*_*m*_ can then be determined via the Arrhenius relationship, as is commonly done for *E*_*A*_. Finally, attempt frequency can be estimated using ν_*D*_ or MD simulations.^27,138,157^ In the argyrodites, Δ*S*_*m*_ values were recently calculated for Li_6–*x*_PS_5–*x*_Br_1–*x*_ via the characteristic frequency approach and Δ*S*_*m*_ was found to positively correlate with Br^–^ occupation on the 4*d* site.^27^ This supports the hypothesis that changes in σ_0_ in Li_6_PS_5_ *X* are driven by changes in Δ*S*_*m*_ that are induced by site disorder, as σ_0_ scales exponentially with site disorder in Li_6_PS_5_ *X* alloys (Figure 8).^18^ As an alternative to the hopping frequency method, Δ*S*_*m*_ was approximated using σ_0_, ν_*D*_ as attempt frequency, and a range of carrier concentrations and jump distances in Li_6_PS_5_(CN)_1–*x*_Br_*x*_.^138^ Validation and routine adoption of these methods would clarify the extent to which Δ*S*_*m*_ or the linear terms drive changes in σ_0_.

### 5.4 The Meyer-Neldel “Compensation” Regime

Just as *E*_*A*_ is generally reduced by softening of the vibrational landscape, σ_0_ is often simultaneously decreased. As discussed above, several terms in σ_0_ remain approximately constant within a material family and the bulk of the changes in σ_0_ can be attributed to Δ*S*_*m*_.^120^ In this context, the simultaneous increase or decrease in *E*_*A*_ and σ_0_ arises from enthalpy–entropy compensation. From the perspective of multiexcitation entropy theory, increasing the height of the activation barrier (*E*_*A*_) means that there are more possible ways to reach the excited state, driving Δ*S*_*m*_ and σ_0_ higher.^120^ This phenomenon is commonly referred to as the Meyer-Neldel compensation rule.^159^ Breaking the Meyer-Neldel compensation rule, or finding materials that follow the so-called anti Meyer-Neldel rule,^160^ is therefore an exciting prospect for achieving high ionic conductivities.

In Li_6_PS_5_ *X* (*X* = Cl^–^, Br^–^, I^–^), the Cl^–^/Br^–^ alloy series is firmly in a “compensation regime”, where changes in *E*_*A*_ and σ_0_ largely cancel one another and ionic conductivity changes very little.^18^ This compensatory behavior is evident when σ_0_ is plotted against *E*_*A*_ (Figure 10), where a linear relationship with positive slope on a logarithmic scale is observed for the Cl^–^/Br^–^ alloy series.^18^ Notably, the I^–^/Br^–^ alloy series does not reside in this regime and can be considered to “break” Meyer-Neldel behavior (Figure 10). Rather, substituting I^–^ with Br^–^ leads to a concurrent decrease in *E*_*A*_ and increase in σ_0_. This results in a three-order-of-magnitude increase in room-temperature ionic conductivity across the alloy series, which is attributed to the introduction of anion site disorder.^18^

**Figure 10:**
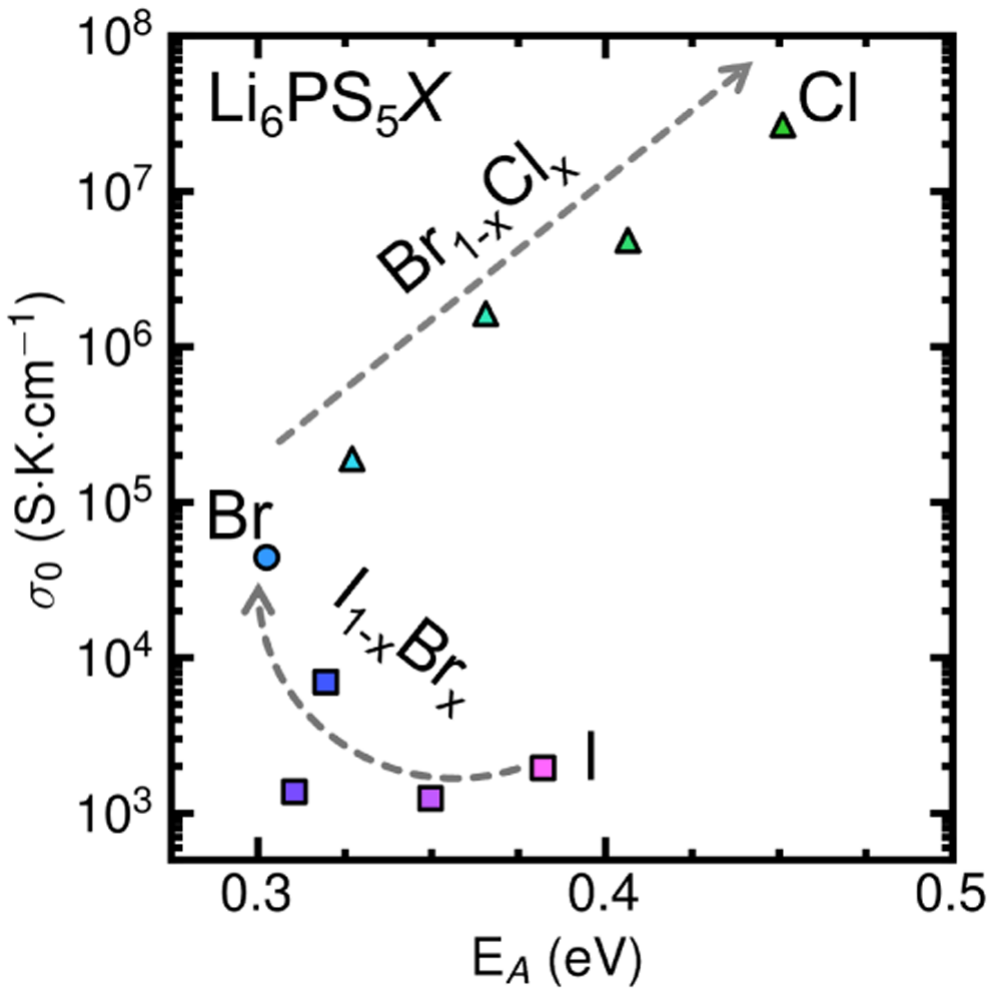
Arrhenius prefactor (σ_0_) plotted against activation energy (*E_A_*) for Li_6_PS_5_ *X* (*X* = Cl^–^, Br^–^, I^–^) and alloys of mixed halides. The introduction of S^2–^/X^–^ site disorder in the Li_6_PS_5_I_1–*x*_Br*_x_* alloy series (pink to blue, squares) causes a simultaneous decrease in activation energy and increase in prefactor, which breaks the Meyer-Neldel rule. Once sufficient disorder is achieved in Li_6_PS_5_Br, the Meyer-Neldel rule holds. This trend is observable in the Li_6_PS_5_Br_1–*x*_Cl*_x_* alloy series (blue to green, triangles). Li_6_PS_5_Br is indicated with a circle as it represents an end member of both alloy series. Gray dashed arrows are guides to the eye indicating the two alloy series and do not represent fits to the data. Data reproduced from ref 18.

Indeed, a variety of argyrodite compositions that do not fall into the compensation regime have been reported (Figure 11). This group of noncompensatory argyrodites is dominated by Li_6+*x*_P_1–*x*_ *M*_*x*_S_5_I, where *M* is a tetravalent cation. As with Br^–^ substitution in Li_6_PS_5_I_1–*x*_Br_*x*_, *M* ^4+^ substitution in Li_6+*x*_P_1–*x*_ *M*_*x*_S_5_I commonly induces S^2–^/I^–^ site mixing, indicating that inducing anion site disorder in anion-ordered argyrodites is an effective approach for inducing noncompensatory behavior.^20,161^ To clarify this design principle: compensatory behavior is expected when site disorder is either present across an entire compositional series or absent across the entire series. In contrast, noncompensatory behavior emerges when site disorder is introduced partway through an alloy series (i.e., when one end member is ordered but intermediate compositions or the opposite end member exhibit disorder). While the loss of site disorder disrupts Meyer-Neldel behavior by localizing the divalent sulfide anion on the 4*d* site, incorporation of high charge density pseudohalides on the 4*d* site produces a similar result. In Li_6_PS_5_(CN)_1–*x*_Br_*x*_, high charge density CN^–^ on the 4*d* site is linked to increased Δ*S*_*m*_ according to eq 3, disrupting Meyer-Neldel behavior.^138^ In these noncompensatory cases, the electrostatics of the 4*d* site play a decisive role in Δ*S*_*m*_. Further discussion of the mechanisms behind this noncompensatory behavior can be found in Section 6. In general, very high ionic conductivities are achieved in soft, polarizable host frameworks that enable anion antisite disorder.

**Figure 11:**
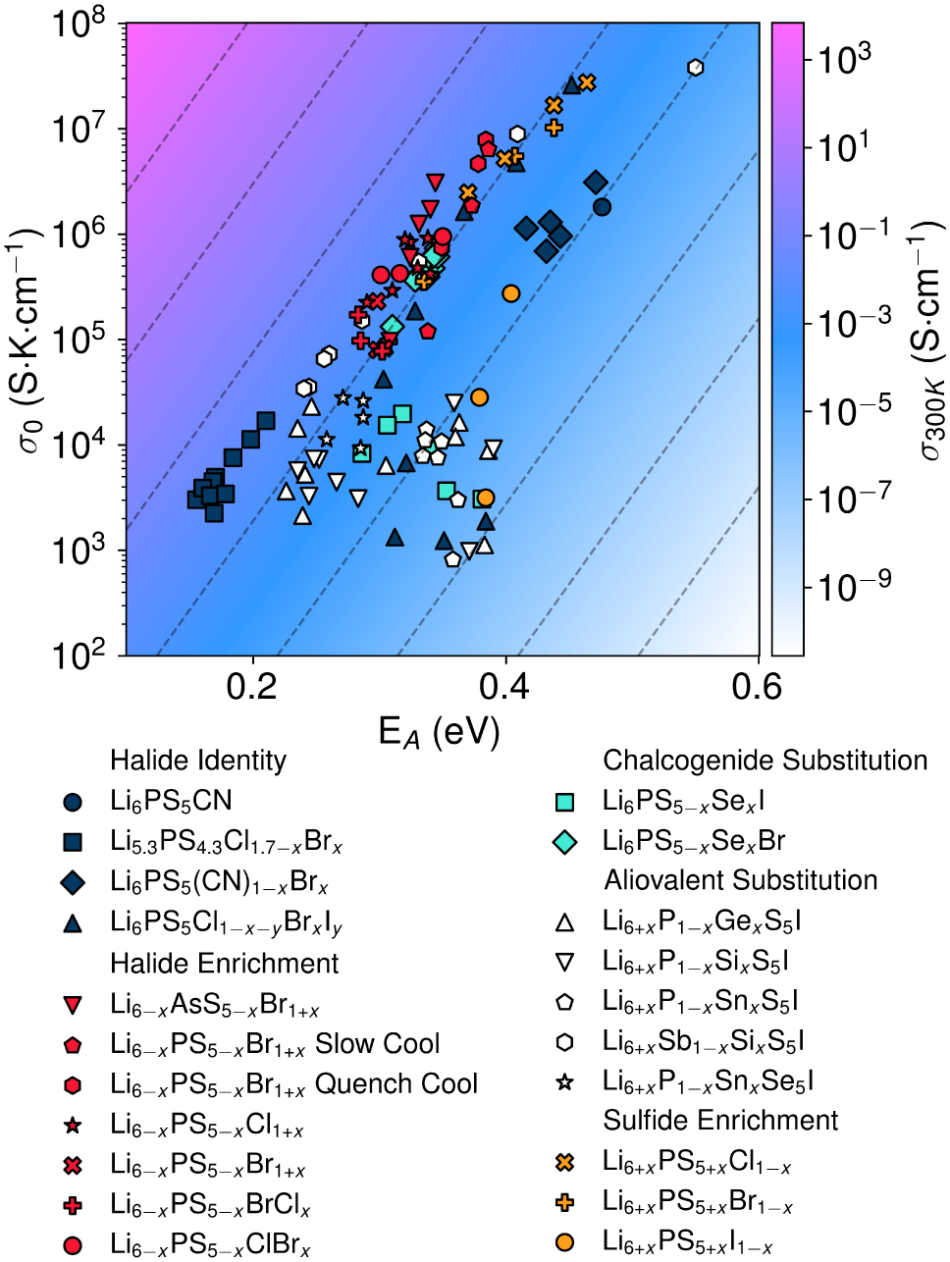
Arrhenius prefactor (σ_0_) plotted against activation energy (*E_A_*) for a selection of lithium argyrodites. The color gradient represents ionic conductivity at *T* = 300 K and the dashed lines represent contours of constant ionic conductivity. Where necessary, σ_0_ was calculated from room temperature ionic conductivity and activation energy according to the Arrhenius relationship with room temperature assumed to be 298 K. Data reproduced from ref 18,20,25,27,28,34–36,50,53,54,108,110,138,154,161 .

## 6 Local Polyanion Dynamics

Argyrodites are amenable to local dynamics of polyhedral and molecular species. In this section, we highlight the known studies of dynamics of the *MCh*_4_ tetrahedral units as well as the dynamics of molecular “pseudohalide” anions and discuss compositional and structural origins that dictate the presence, timescales, and amplitudes of these dynamics. Given the presence of these dynamics, we then discuss potential mechanisms for coupling between polyanion motion and Li^+^ diffusion.

### 6.1 Polyhedral Dynamics

The structurally isolated *MCh*_4_ units can undergo dynamics, and the timescales of these motions are sensitive to the surrounding environment. ^31^P spin lattice relaxation nuclear magnetic resonance (SLR NMR) studies have recently provided extensive insights into ${\mathrm{PS}}_{4}^{3-}$ dynamics in the lithium halide argyrodites (Table 2).

**Table 2:** Time Constants (*τ*_0_, s) and Activation Energies (*E_A_*, eV) for ${\mathrm{PS}}_{4}^{3-}$ and Li^+^ Motion for Several Lithium Halide Argyrodites, as Determined by ^31^P and ^7^Li Spin-Lattice Relaxation Solid-State NMR Studies in the Laboratory Frame^a^

Compound	^31^P τ_0_ (s)	^7^Li τ_0_ (s)	^31^P *E*_*A*_ (eV)	^7^Li *E*_*A*_ (eV)	*T*_*max*_ (K)	Note	Reference(s)
Li_6_PS_5_Cl	5.00 × 10^–13^	1.30 × 10^–14^	0.18	0.32	283	^31^P peak due to ${\mathrm{PS}}_{4}^{3-}$ rotations and nearby Li^+^ diffusion	^49^
Li_6_PS_5_Br	9.09 × 10^–13^	2.27 × 10^–14^	0.15	0.21	233	^31^P low *T* peak due to ${\mathrm{PS}}_{4}^{3-}$ rotations	^49^
Li_6_PS_5_Br	9.09 × 10^–12^	2.27 × 10^–14^	0.14	0.21	–	^31^P high *T* peak due to nearby Li^+^ diffusion	^49^
Li_6_PS_5_I	5.55 × 10^–14^	8.33 × 10^–13^	0.2	0.2	223	^31^P low *T* peak due to ${\mathrm{PS}}_{4}^{3-}$ rotations	^49^
Li_6_PS_5_I	3.45 × 10^–11^	8.33 × 10^–13^	0.18	0.2	–	^31^P high *T* peak due to nearby Li^+^ diffusion	^49^
Li_6_PS_5_I	3.45 × 10^–12^	8.33 × 10^–13^	0.18	0.2	–	Peak 1; translational, fast Li^+^ intracage dynamics	^131^
Li_6_PS_5_I	5.00 × 10^–12^	3.33 × 10^–13^	0.44	0.26	–	Peak 2; translational, fast Li^+^ exchange between cages	^131^
Li_6_PS_5_I	5.55 × 10^–14^	–	0.2	–	220	Peak 3; rotational jumps of the polyanions	^131^
Li_6_PS_5_I	4(3) × 10^–12^	0.8(1) × 10^–12^	0.44(4)	0.18(1)	219		^24^
Li_6.1_P_0.9_Ge_0.1_S_5_I	7(3) × 10^–11^	1.5(8) × 10^–12^	0.29(2)	0.119(3)	230		^24^
Li_6.3_P_0.7_Ge_0.3_S_5_I	4(1) × 10^–9^	4(2) × 10^–12^	0.17(1)	0.098(1)	236		^24^
Li_6.6_P_0.4_Ge_0.6_S_5_I	7(4) × 10^–10^	0.8(5) × 10^–12^	0.13(1)	0.119(4)	253		^24^

a *T*_*max*_ corresponds to the temperature at which the rotational correlation rate (1/*τ*_*rot*_) for ${\mathrm{PS}}_{4}^{3-}$ rotations is ∼10^9^ s^–1^ as determined by the rate peak of ^31^P SLR measurements (Larmor frequency of 121 MHz).

Early studies found that the identity of the halide anion strongly correlates with the timescales of ${\mathrm{PS}}_{4}^{3-}$ dynamics. Across the series Li_6_PS_5_Cl → Li_6_PS_5_Br → Li_6_PS_5_I, introducing larger and more polarizable halides results in a progressive increase in the rotation rates of the ${\mathrm{PS}}_{4}^{3-}$ tetrahedra (Figure 12).^49^ Li_6_PS_5_Br and Li_6_PS_5_I both exhibit two distinct types of relaxation processes, which have been assigned to ${\mathrm{PS}}_{4}^{3-}$ dynamics (low *T* peak) and relaxation due to the transit of nearby Li^+^ spins (high *T* peak).^49,131^ In Li_6_PS_5_Cl, only a single feature is observed, indicating that both relaxation processes are occurring on similar time scales. In SLR NMR, the probed rate is fixed by the Larmor frequency (ω_0_) of the probed nucleus and temperature is varied; when a peak in log­(1/*T*_1_) versus 1/*T* is observed, the correlation rate of the observed process (1/τ_*rot*_) is equal to 2π × ω_0_. Rate peaks corresponding to ${\mathrm{PS}}_{4}^{3-}$ rotations occurring at ∼10^9^ s^–1^ (ω_0_ = 121 MHz) are observed at *T* = 283, 233, and 223 K for Li_6_PS_5_Cl, Li_6_PS_5_Br, and Li_6_PS_5_I, respectively (Table 2).^49^ The systematic shift of the rate peak to lower temperatures is indicative of faster ${\mathrm{PS}}_{4}^{3-}$ rotational dynamics.

**Figure 12:**
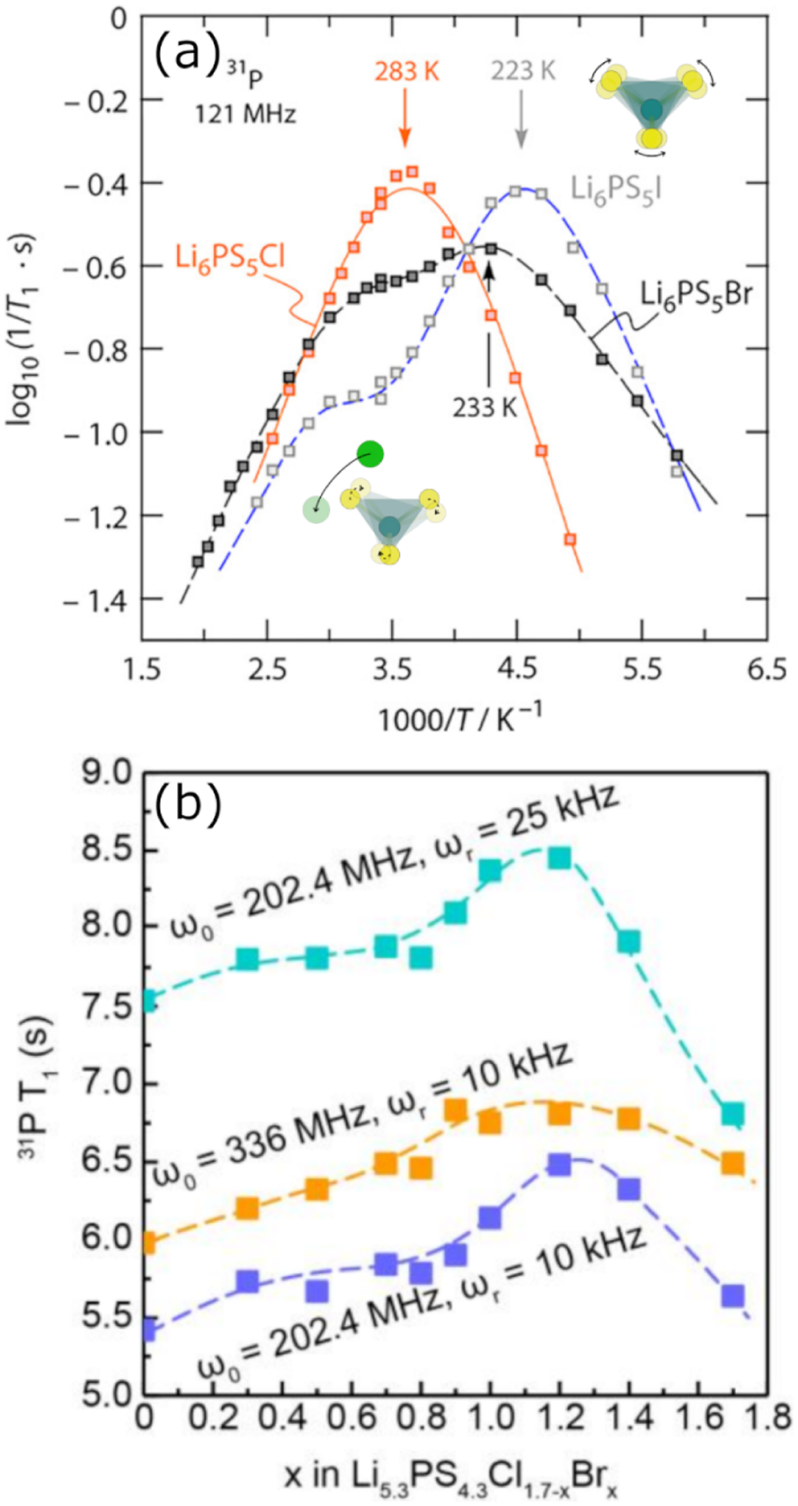
(a) ^31^P spin–lattice relaxation nuclear magnetic resonance (SLR NMR) provides insight into ${\mathrm{PS}}_{4}^{3-}$ rotational dynamics. In Li_6_PS_5_I and Li_6_PS_5_Br, the lower temperature rate peaks are attributed to ${\mathrm{PS}}_{4}^{3-}$ rotations (right inset), while the higher temperature shoulders are associated with Li^+^ hopping sensed by ^31^P spins (left inset). Peaks at lower temperatures correspond to faster processes. The two features cannot be distinguished in Li_6_PS_5_Cl, which indicates that the two processes occur at comparable rates. (b) ${\mathrm{PS}}_{4}^{3-}$ tetrahedra exhibit faster dynamics for mixed halide compositions, due to the extensive anion site disorder that creates a distribution of local environments for the tetrahedral units. (a) adapted and (b) reproduced with permission from ref 49 and ref 36. Copyrights 2019 American Chemical Society and 2023 John Wiley and Sons, respectively.

In the same way that anion antisite disorder strongly impacts lithium ion dynamics (Section 4), anion disorder also impacts the timescales of tetrahedral rotations. A ^31^P SLR study of the halide series Li_5.3_PS_4.3_Cl_1.7–*x*_Br_*x*_ series demonstrated that Li^+^ and ${\mathrm{PS}}_{4}^{3-}$ exhibit similar motional rates in heavily halide-enriched compositions, just as in Li_6_PS_5_Cl.^36,49^ Increasing bromide content leads to faster timescales of ${\mathrm{PS}}_{4}^{3-}$ dynamics, but does not impact rotation rates sufficiently to yield distinct timescales for ${\mathrm{PS}}_{4}^{3-}$ and Li^+^ motions.^36^ Mixed-halide members of the halide-rich compositional series appear to exhibit faster ${\mathrm{PS}}_{4}^{3-}$ dynamics than either of the end-members. Faster tetrahedral dynamics were also observed when the local anion configuration favored Cl^–^, Br^–^, and S^2–^ site sharing on the 4*d* site compared to when the 4*d* site was occupied by S^2–^ and one halide species.^36^ Taken together, these observations suggest that anion site disorder creates a broader variety of local environments experienced by the tetrahedral units, and that reducing the average anion charge and charge density of the 4*d* site enables faster tetrahedral rotations. Notably, changes in rotation rate for the Li_5.3_PS_4.3_Cl_1.7–*x*_Br_*x*_ series were not reported to cause ${\mathrm{PS}}_{4}^{3-}$ and Li^+^ motions to occur on differing time scales, indicating that the impacts of site disorder and halide identity in the very halide-rich regime are small in comparison to those observed from Li_6_PS_5_Cl → Li_6_PS_5_Br → Li_6_PS_5_I.^36,49^

The timescales of tetrahedral dynamics are also sensitive to cation substitution effects at the center of the tetrahedra. Ge^4+^ substitution in the Li_6+*x*_P_1–*x*_Ge_0.1_S_5_I series is accompanied by slowing of the ${\mathrm{PS}}_{4}^{3-}$ dynamics.^24^ This may be due to differences in cation radii (*r*_*P*_ = 0.17 Å, *r*_*Ge*_ = 0.39 Å),^162^ introduction of anion site disorder, or the larger average anion charge of the ${\mathrm{GeS}}_{4}^{4-}$ tetrahedra that modify the environment in the vicinity of the ${\mathrm{PS}}_{4}^{3-}$ units. ^31^P relaxometry measurements are selective for the phosphorus nuclei, and thus the dynamics of the ${\mathrm{GeS}}_{4}^{4-}$ tetrahedra remain an open question. As ^31^P relaxometry studies of cation-substituted argyrodites are sparse, further studies are needed to establish how the composition of the tetrahedra impacts their dynamics.

Signatures of (dynamic) rotational disorder of the isolated tetrahedral units have been observed through static, local structure probes. As early as 2017, experimental pair distribution functions (PDF) of Li_6_PS_5_ *X* (*X* = Cl^–^, Br^–^, I^–^) revealed inconsistencies between the local structure and the *F*4̅3*m* average structure observed by Bragg diffraction.^18^ Modeling methods that capture this disorder have recently enabled quantification of the spatial extent of tetrahedral dynamics in the argyrodites.^38,40^ Recently, we employed a rigid-body modeling routine in which the ${\mathrm{PS}}_{4}^{3-}$ units were allowed to rotate freely.^38^ The resulting supercell exhibits pseudorandom rotations of the ${\mathrm{PS}}_{4}^{3-}$ away from their equilibrium positions by angles of ∼20–30° (Figures 13 and 14). By quantifying the distribution of displacements relative to the cubic structure, we found that the amplitude of the rotations increase across the Li_6_PS_5_I < Li_6_PS_5_Br < Li_6_PS_5_Cl series and are strongly correlated with site disorder (Figure 14). Given this local structure information and the aforementioned NMR studies, ${\mathrm{PS}}_{4}^{3-}$ rotation rates appear to be inversely correlated with rotation amplitude and 4*a*/4*d* site mixing; Li_6_PS_5_I exhibits the fastest and smallest rotations, whereas Li_6_PS_5_Cl exhibits the slowest and largest. Notably, the neutron PDF of Li_6_PS_4.5_Se_0.5_Br reveals similar signatures of tetrahedral rotational disorder; however, the deviations appear to be significantly less with Se^2–^ than with S^2–^. This observation suggests that the larger Se^2–^ anion modifies the amplitudes and timescales of polyhedral rotations.^154^

**Figure 13:**
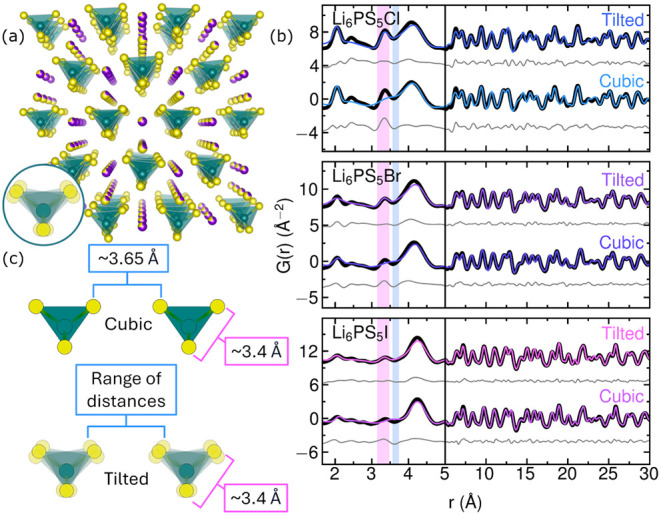
Dynamic tilting of the ${\mathrm{PS}}_{4}^{3-}$ tetrahedra (a) in Li_6_PS_5_ *X* (*X* = Cl^–^, Br^–^, I^–^) leads to discrepancies between the local and average structures (b).^38^ As illustrated in (b), fitting with the cubic *F*4̅3*m* average structural model results in undermodelling of the intra- ${\mathrm{PS}}_{4}^{3-}$ S-S pair correlation (∼3.4 Å, pink vertical bar) and overmodelling of the inter- ${\mathrm{PS}}_{4}^{3-}$ S-S pair correlation (∼3.65 Å, blue vertical bar). Allowing ${\mathrm{PS}}_{4}^{3-}$ units to rotate as rigid bodies results in a range of inter- ${\mathrm{PS}}_{4}^{3-}$ S-S distances and improves fitting of the highlighted pair correlations. This behavior is shown in (c), where the pink callouts correspond to the intra- ${\mathrm{PS}}_{4}^{3-}$ S-S distances and the blue callouts correspond to the inter- ${\mathrm{PS}}_{4}^{3-}$ S-S distances. Data reproduced from ref 38.

**Figure 14:**
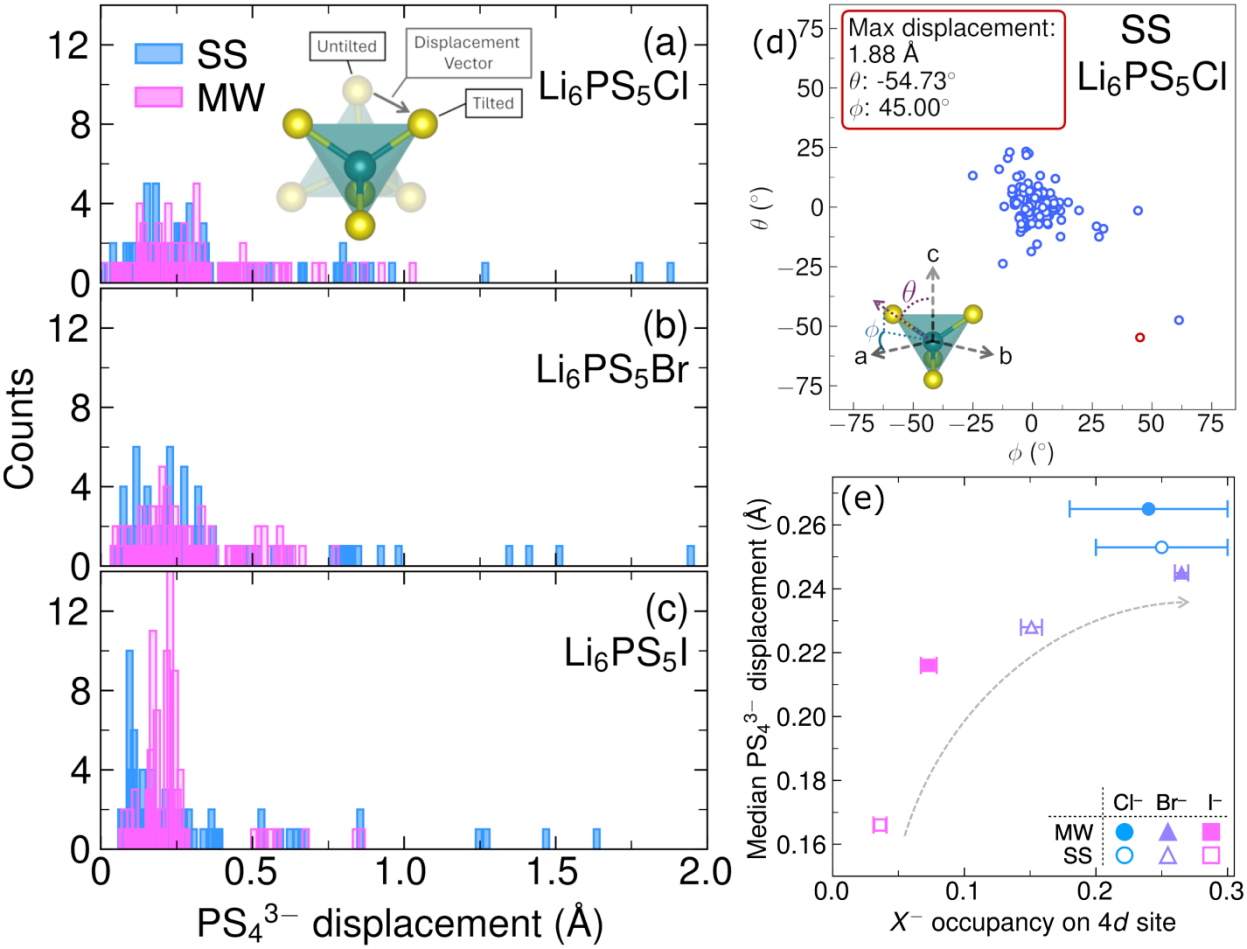
Rotational displacements of ${\mathrm{PS}}_{4}^{3-}$ units are correlated with halide identity, synthesis method, and S^2–^/*X* ^–^ site mixing. Panels (a), (b), and (c) show histograms of polyhedral rotational displacement as a function of synthesis method. SS corresponds to materials prepared by traditional solid-state synthesis and MW corresponds to materials prepared by rapid microwave synthesis method. The directionality and magnitude of polyhedral displacements is pseudorandom and clustered about the ${\mathrm{PS}}_{4}^{3-}$ orientation in the *F*4̅3*m* aristotype (d). ${\mathrm{PS}}_{4}^{3-}$ displacement is positively correlated with S^2–^/*X* ^–^ site mixing (e). Adapted with permission from ref 38. Copyright 2025 John Wiley and Sons.

The lithium ion transport properties of the Li_6_PS_5_ *X* (*X* = Cl^–^, Br^–^, I^–^) argyrodites are correlated with the amplitude and timescales of tetrahedral dynamics. As shown in Figure 15, higher median amplitudes of the ${\mathrm{PS}}_{4}^{3-}$ rotations determined by X-ray PDF (XPDF) are correlated with lower activation barriers (*E*_*A*_), larger Arrhenius prefactors (σ_0_) and generally higher ionic conductivities (σ). Lower activation energies track with both the degree of site disorder and higher amplitudes of tetrahedral rotations. As discussed in Section 4, anion site disorder creates a broader distribution of low-energy pathways for Li^+^, which contribute to lower activation energies for macroscopic transport (Figure 5).^135^ Larger-amplitude displacements of the ${\mathrm{PS}}_{4}^{3-}$ are expected to further contribute to this effect by creating dynamically fluctuating bottleneck areas for mobile ions. Expanding the bottleneck area through which ions must pass generally decreases *E*_*A*_ by reducing the electrostatic interactions between the mobile ion and the anion sublattice.^117,163–165^ This phenomenon is often interpreted through a static structural picture,^20,110,154,161^ but polyanion rotations^47,166–168^ and translations^97^ as well as “dynamic breathing” of the lattice^149^ are known to transiently change bottlenecks and facilitate ion transport. The exponential increase in σ_0_ with increasing polyhedral displacement amplitudes suggests that the dynamics of the tetrahedra modify the vibrational properties surrounding mobile ion sites. Assuming quasi-random librations of all polyhedra in the argyrodite structure, changes in oscillation rate and amplitude disproportionately affect the vibrational environments of the stable sites (T5, T5a) and the saddle points (T5-T2, T2-T2, and T5-T4 shared faces) relevant to ion conduction. These sites are vibrationally distinct because their geometric relationships to the tetrahedral units differ (Figure 15c and d). Consequently, modifications to the overall oscillatory behavior of the polyhedra alter the vibrational partition functions of the initial state and saddle point, increasing Δ*S*_*m*_ (eq 3) and, in turn, σ_0_ (Figure 15e).

**Figure 15:**
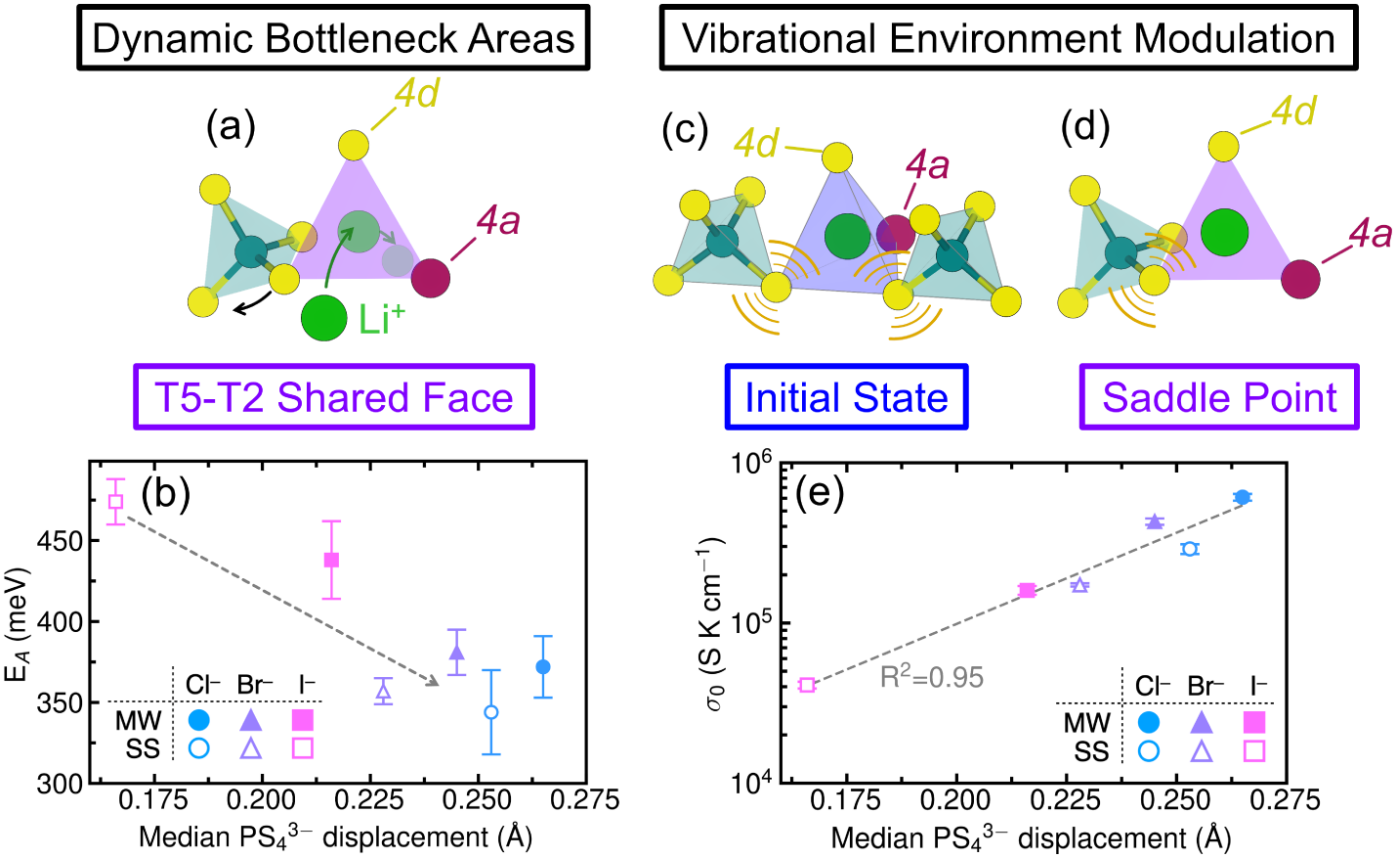
T5-T2 shared face represents a likely bottleneck for ion conduction in Li_6_PS_5_ *X* argyrodites. It is defined by a 4*d* site, a 4*a* site, and one bonded tetrahedral sulfur. Polyhedral librations dynamically change the bottleneck area (a), facilitating Li^+^ hops through the bottleneck. (b) illustrates the resulting dependence of *E_A_* on polyhedral displacement. Changes in polyhedral rotational behavior disproportionately impact the vibrational environment about the initial state (c) and the saddle point (d). This results in a strong dependence of σ_0_ upon polyhedral displacement, as shown in (e). Filled symbols (MW) correspond to argyrodites synthesized via a dry microwave method, while open symbols (SS) represent materials made via a conventional solid-state route. Polyhedral displacement is defined as the distance between a bound sulfur in the *F*4̅3*m* structure and the position of that sulfur in the rotationally disordered structure. Note that the T5-T2 hop is assumed to be rate limiting for the purposes of this figure, although the T2-T2 and T5-T4 hops may also represent rate limiting steps for intercage hopping. Panels (b) and (e) adapted with permission from ref 38. Copyright 2025 John Wiley and Sons.

The presence of dynamic polyhedral rotations holds additional implications for the phase transition behavior observed in the argyrodites. Order–disorder transitions in materials with dynamic polyhedral rotations are often driven by softening and eventual freezing out of rotary modes with decreasing temperature.^169,170^ A recent PDF study in Ag_8_GeS_6_, Ag_9_GaSe_6_, and Ag_8_GeTe_6_ argyrodites revealed that the low-r pair correlations of the high temperature aristotype were better modeled by a hettotype structure, indicating that disordering occurs concurrent with the phase transition.^39^ Therefore, we hypothesize that activation of libratory modes—likely through dynamic sampling of local hettotype configurations—drives order–disorder phase transitions in argyrodites. As discussed in Sections 5 and 2, the phase transitions are accompanied by significant disordering of the mobile ion sublattice, which may be further impacted by the onset of polyhedral dynamics.^29,33,39^ A closely related phenomenon is observed in Na_3_PS_4_, where the α → β phase transition is accompanied by activation of ${\mathrm{PS}}_{4}^{3-}$ dynamics that facilitate fast ion transport.^41^ In both Na_3_PS_4_ and the substituted analog Na_3–*x*_Sb_1–*x*_W_*x*_S_4_, the XPDF of the high-temperature phase retains signatures of the lower-symmetry hettotype characterized by tilted *M*S_4_ tetrahedra.^41,42,171^ Taken together, evidence from the argyrodites and the Na_3_PS_4_ structural family points toward a common mechanistic motif: order–disorder phase transitions characterized by activation of polyhedral dynamics that simultaneously promote fast ion transport.

### 6.2 Molecular Moieties

Beyond ${\mathrm{PS}}_{4}^{3-}$ units, the polyatomic pseudohalides ${\mathrm{BH}}_{4}^{-}$ and CN^–^ can be substituted at the halide position(s) in Li_6_PS_5_ *X*.^105,107,108,138,172^ Substituting molecular species at these sites introduces new rotational and vibrational dynamics into the argyrodite framework.

Incorporation of tetrahedral ${\mathrm{BH}}_{4}^{-}$ in Li_6_PS_5_BH_4_ represents one approach to add additional polyhedral anions to the lattice, but synthesizing the target phase has proven challenging. Formation of the argyrodite phase requires LiBH_4_-rich precursors.^105,106^ This stems from the thermal instability of LiBH_4_, which decomposes at typical synthesis temperatures for Li_6_PS_5_ *X*; reliable reports of Li_6_PS_5_BH_4_ are therefore scarce.^173^ Nevertheless, Li_6_PS_5_BH_4_ is reported to exhibit higher ionic conductivity than both Li_6_PS_5_Cl and Li_6_PS_5_Br.^105^ Computational studies disagree on the atomistic origin of this enhancement: some report that ${\mathrm{BH}}_{4}^{-}$ rotates responsively to Li^+^ hops and thereby facilitates diffusion,^172^ whereas others suggest that weak Li-H interactions, rather than ${\mathrm{BH}}_{4}^{-}$ reorientation, are responsible.^105^ The possibility of coupled rotational dynamics between ${\mathrm{PS}}_{4}^{3-}$ and ${\mathrm{BH}}_{4}^{-}$ remains largely unexplored. We note that H^+^ conduction may be nontrivial due to incorporation of H^+^ liberated from excess LiBH_4_, but this has not been thoroughly investigated.^105,172,173^ If H^+^ is present and mobile in as-prepared Li_6_PS_5_BH_4_, the elevated ionic conductivity observed in Li_6_PS_5_BH_4_ could be an outgrowth of H^+^ transport rather than enhanced Li^+^ conduction.

Li_6_PS_5_CN represents an interesting case due to its dipolar pseudohalide—where both ${\mathrm{PS}}_{4}^{3-}$ and ${\mathrm{BH}}_{4}^{-}$ are tetrahedral, CN^–^ adopts a linear geometry. As such, the arrangement of CN^–^ ions in Li_6_PS_5_CN exhibits a strong influence on the presence and nature of their dynamics. Initial analysis of Li_6_PS_5_CN found that CN^–^ was distributed across the 4*a* and 4*d* Wyckoff positions and was orientationally disordered.^107^ This led to the hypothesis that CN^–^ may undergo dynamic rotations that allow for a lowered activation energy for Li^+^ hopping relative to Li_6_PS_5_Br.^107^ Further molecular dynamics investigations revealed that the rotational dynamics of CN^–^ anions are highly dependent on S^2–^/CN^–^ site mixing, with greater degrees of site mixing corresponding to slower and more constrained rotational dynamics, such as “wobbling in a cone″ motions (Figure 16).^108^ This phenomenon is hypothesized to be driven by CN^–^-CN^–^ elastic dipole coupling. In ordered configurations with no S^2–^/CN^–^ disorder, each CN^–^ is ∼7 Å from its nearest CN^–^ neighbor.^108^ However, in configurations with S^2–^/CN^–^ site mixing, the CN^–^-CN^–^ distances are reduced, which increases elastic dipole coupling and increases the energetic cost for rotations.^108^

**Figure 16:**
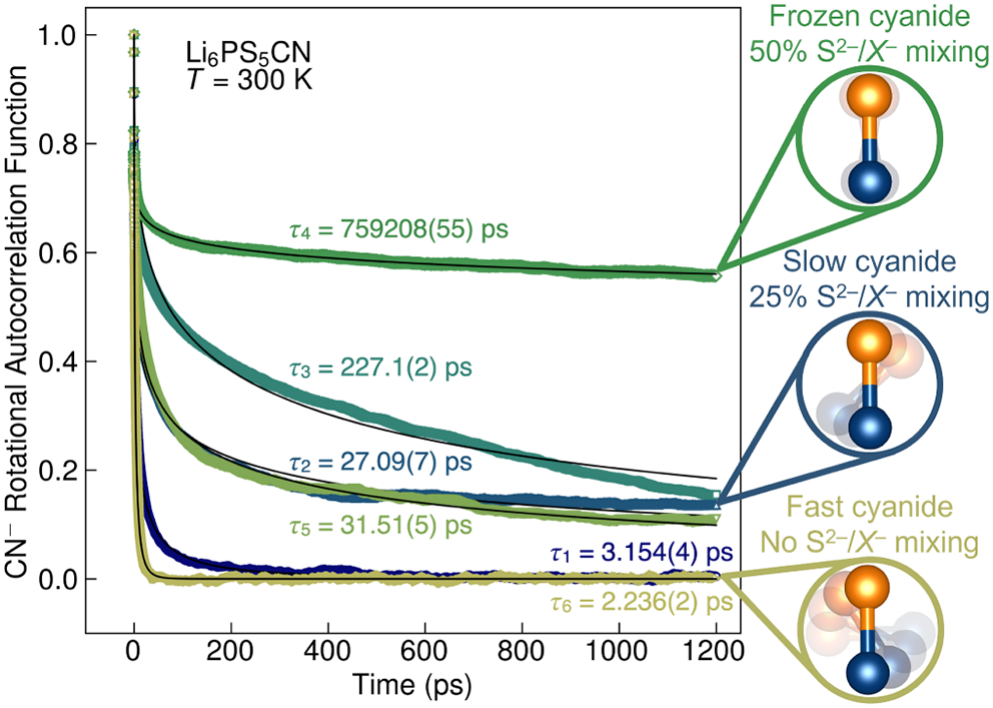
Rotational autocorrelation function for cyanide ions calculated for different configurations of anion site disorder. MD simulations were performed at *T* = 300 K. For fully ordered Li_6_PS_5_CN, rapid tumbling is observed on the picosecond time scale (τ_1_, τ_6_). Introduction of 25% anion disorder slows rotational dynamics by an order of magnitude (τ_2_, τ_5_) and inhibits full rotations, presumably favoring oscillatory or wobbling behaviors. At 50% anion site mixing, rotational dynamics occur on timescales ranging from hundreds to hundreds of thousands of picoseconds, indicating that rotational dynamics are significantly slowed and constrained to libratory behavior. Adapted from ref 108. Copyright 2024 American Chemical Society.

The apparent tunability of CN^–^ dynamics based on site disorder may provide an opportunity to investigate the influence of rotationally active pseudohalides upon ${\mathrm{PS}}_{4}^{3-}$ dynamics. The time scales and nature of CN^–^ rotations can be tuned via manipulating S^2–^/CN^–^ site disorder, which can be modulated via alloying with a halide or through varying the S^2–^/CN^–^ ratio.^108,138^ The potential to activate or deactivate CN^–^ rotational dynamics may allow for a systematic study of how CN^–^ rotational disorder influences ${\mathrm{PS}}_{4}^{3-}$ dynamics. In Li_6_PS_5_BH_4_, even the nature of ${\mathrm{BH}}_{4}^{-}$ rotational behavior remains unclear. Some open questions in this area include:

- How do the presence and timescales of pseudohalide dynamics impact rotational motions of larger tetrahedral units, and vice versa?
- To what extent do pseudohalide rotations couple to neighboring thiophosphate units, lithium ions, or both?
- Do uncoupled rotations lead to an enhancement of the dynamic bottleneck areas and vibrational environment modifications discussed in Section 6.1?

### 6.3 Coupled Dynamics

The proclivity for argyrodites to exhibit rotational disorder of polyhedral and molecular species (Sections 6.1 and 6.2) motivates the possibility for *coupled dynamics* between rotationally active species and ion migration. Polyhedral and molecular rotations have long been thought to facilitate ion transport; the extent of coupling and underlying mechanisms vary across material families and has been the subject of controversy since the 1980s.^47,171,174–181^ The “paddlewheel” mechanism is characterized by large-amplitude rotations of the polyhedral species that are proposed to *actively* couple to Li^+^ hopping (Figure 17a).^47,179^ In contrast, a responsive “soft cradle” mechanism has been proposed, in which polyhedral or molecular units reorient to accommodate ion motion (Figure 17b).^178^ A third proposed mechanism involves a gate or percolation model where vibrations and rotations of polyanions are not coupled with cation hopping, but transiently open low-energy conduction pathways through which ions can transit.^47,167,168,182,182^ Although the precise mechanisms remain controversial, there is consensus that the dynamics of polyhedral or molecular reorientations are generally beneficial for ion transport.^47,180,181^

**Figure 17:**
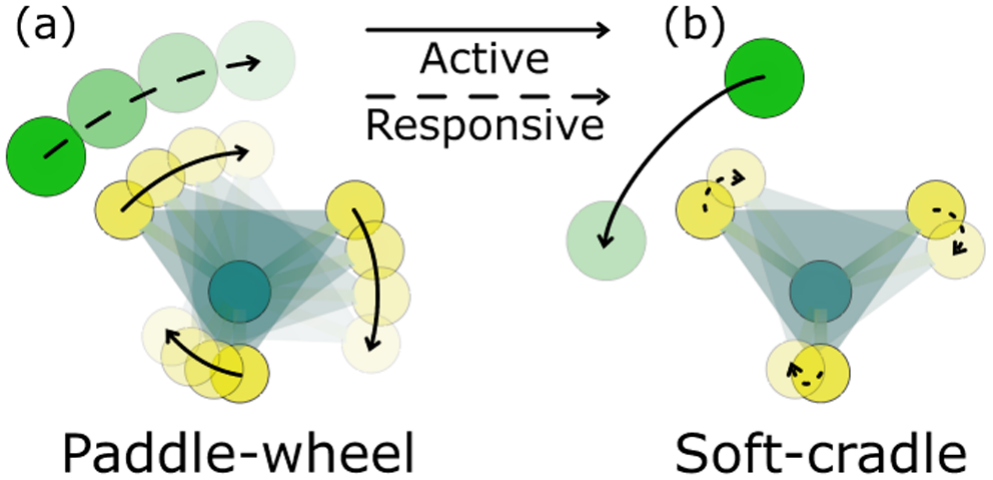
Illustration of two proposed mechanisms by which ${\mathrm{PS}}_{4}^{3-}$ rotations can couple directly to Li^+^ dynamics. In the paddle-wheel mechanism (a), active rotations of ${\mathrm{PS}}_{4}^{3-}$ facilitate responsive Li^+^ transport. In the soft-cradle mechanism (b), ${\mathrm{PS}}_{4}^{3-}$ rotates in response to Li^+^ hops.

The relative timescales of rotational dynamics and mobile ion hopping provide insight into the potential for coupled dynamics.^49,178,179,183^ As discussed above, the argyrodites may exhibit dynamics of both polyhedral and molecular species; the timescales of these motions are sensitive to the local environment. ${\mathrm{PS}}_{4}^{3-}$ rotations in Li_6_PS_5_Cl occur at a rate of approximately 10^9^ s^–1^ at *T* = 283 K, while Li_6_PS_5_Br and Li_6_PS_5_I reach this rate at *T* = 233 K and *T* = 223 K, respectively.^49^ Molecular dynamics (MD) simulations indicate that Li^+^ hop *transit times* (τ_*hop*_)—the time from the beginning to end of an individual successful hop—are on the order of 10^–12^ s.^22,149^ Thus, polyhedral rotational dynamics occur roughly 3 orders of magnitude more slowly than *individual* Li^+^ hops, indicating that polyhedral dynamics are temporally decoupled from individual Li^+^ hops. Similarly, lithium and cyanide dynamics occur on distinct timescales in the cyanide argyrodite Li_6_PS_5_CN; according to MD simulations, anion-ordered structures enable faster cyanide rotational dynamics (∼10^–12^ s) and yield slower Li^+^ dynamics (∼10^–9^ s), while anion-disorder results in fast Li^+^ hopping (∼10^–11^ s) but slow or frozen cyanide rotations (<10^–7^ s).^108^

In the case of Li_6_PS_5_Cl and Li_6_PS_5_Br, ${\mathrm{PS}}_{4}^{3-}$ rotation rates (1/τ_*rot*_) approach the rate of *successful* Li^+^ hopping (1/τ_*suc*_; ≈ 10^9^ s^–1^), which may indicate Li^+^- ${\mathrm{PS}}_{4}^{3-}$ coupling (Figure 12).^49,125,131^ It is important to note that τ_*suc*_ ≠ τ_*hop*_; 1/τ_*suc*_ corresponds to the rate at which successful hopping events occur, while τ_*hop*_ is the amount of time it takes for a single successful hop to occur from beginning to end. The similar values of 1/τ_*rot*_ and 1/τ_*suc*_ indicate that Li^+^ jump attempts are successful on the same time scale that ${\mathrm{PS}}_{4}^{3-}$ units rotate and ${\mathrm{PS}}_{4}^{3-}$ units may be *causally implicated* in those jumps. The much faster time scale of τ_*hop*_ than τ_*rot*_ indicates that individual hops are completed far more quickly than polyhedral rotations, implying that the two processes are *temporally decoupled*.

Computational investigations of the argyrodites indicate that *MCh*_4_ dynamics strongly influence ion transport. A recent study on Li_6_PS_5_Cl indicated that freezing ${\mathrm{PS}}_{4}^{3-}$ units in place was deleterious to ion transport, but that allowing them to translate and rotate effectively recovered ion diffusion properties.^149^ Further, simulations in which ${\mathrm{PS}}_{4}^{3-}$ units are allowed to rotate about their central phosphorus indicate that these rotations nearly double Li^+^ diffusivity in Li_6_PS_5_Cl relative to the static ${\mathrm{PS}}_{4}^{3-}$ control.^184^ However, ${\mathrm{PSe}}_{4}^{3-}$ translational rather than rotational dynamics appear to be the main factor correlated with Cu^+^ mobility in aristotypic Cu_7_PSe_6_.^97^ Although ${\mathrm{PSe}}_{4}^{3-}$ rotations were not considered crucial in Cu^+^ transport, no simulations in which ${\mathrm{PSe}}_{4}^{3-}$ rotations were allowed without translational freedom were performed.^97^ Cooperative translational-rotational motions may serve to dynamically modulate ion transport bottlenecks or actively shuttle ions through the lattice.

The timescales and amplitudes of polyhedral rotations in Li_6_PS_5_ *X* suggest a combination of gate-like modification of ion transport pathways and soft-cradle accommodation of Li^+^. Systems in which polyhedral rotations significantly exceed the upper bound for librations (∼30°) are often considered candidates for paddlewheel dynamics, although some studies define full 120° *C*_3_ tetrahedral rotations as necessary in ${\mathrm{PS}}_{4}^{3-}$ -containing systems.^178,179^ From local structure studies of Li_6_PS_5_ *X*, the ${\mathrm{PS}}_{4}^{3-}$ tetrahedra exhibit relatively low-amplitude librations with rotation angles < 25° (Figure 14d) that are insufficient for a paddlewheel mechanism.^178^ Rather, we propose that the relatively low-angle polyhedral rotations where the rotation rate is slower than or on the same order as ion hopping processes give rise to widened ion transport pathways that persist long enough for mobile ions to traverse the relevant bottlenecks (Figure 15a and b). This mechanism is reminiscent of the proposed “gate action” in Li_2_SO_4_, where ${\mathrm{SO}}_{4}^{2-}$ opens and closes ion conduction pathways as it undergoes rotations.^47,182^ This gate-type mechanism most likely operates in tandem with a soft-cradle effect; the equilibrium orientations about which ${\mathrm{PS}}_{4}^{3-}$ units oscillate shift to accommodate local cation arrangements, consistent with computational studies that suggest translational and rotational dynamics contribute to transport.^97,149,184^ This proposed mechanism can be considered pseudostatic—so long as the lifetime of the widened bottleneck (τ_*open*_) exceeds the time required for an ion to traverse it (τ_*hop*_), the effective bottleneck area governing transport is the maximum area sampled as the polyhedral units librate. In this limit (τ_*open*_ ≫ τ_*hop*_), transient structural distortions contribute to transport in much the same way as static bottleneck expansion. This pseudostatic picture is supported by the observation that polyhedral rotational dynamics occur roughly 3 orders of magnitude more slowly than individual Li^+^ hops.^49,149^

Although NMR relaxometry highlights differences in rotation *rates* across Li_6_PS_5_ *X* compositions, our pseudostatic view implies that *amplitude*, rather than frequency, is the transport-relevant variable. Our recent work demonstrated that ${\mathrm{PS}}_{4}^{3-}$ rotational displacements increased across the Li_6_PS_5_I → Li_6_PS_5_Br → Li_6_PS_5_Cl series.^38^ Taken together, these results suggest that the tetrahedra in Li_6_PS_5_I undergo rapid but shallow oscillations, producing small bottleneck fluctuations, while those in Li_6_PS_5_Cl undergo slower and larger oscillations that more substantially widen ion-transport pathways, with intermediate behavior in Li_6_PS_5_Br. Because τ_*open*_ ≫ τ_*hop*_, differences in rotation rates are unlikely to disrupt the pseudostatic picture. Rather, the larger rotational amplitudes in Li_6_PS_5_Br and Li_6_PS_5_Cl are sufficient to lower *E*_*A*_, while the fast but shallow rotations in Li_6_PS_5_I inhibit transport and yield higher *E*_*A*_ values. The proposed “gate” mechanism is also consistent with the well-established design principle that anion antisite disorder lowers the energetic barriers for intercage hopping. Anion site disorder is associated with larger-amplitude tetrahedral rotations that widen the bottlenecks for ion hopping. Coupled with the electrostatic averaging picture described in Section 4, this observation may explain why relatively small degrees of anion site disorder (<10%) can have large impacts on macroscopic ionic conductivity.^161^

Given the shared structural and dynamic features between argyrodites and other materials systems comprised of dynamic polyhedral species, further investigation of libration-assisted transport and associated phase behavior is warranted. Several open questions that may clarify the underlying mechanisms are outlined below:

- Do polyanion librations occur independently of cation hopping—merely opening enthalpically and entropically favorable diffusion pathways—or do cation hops actively induce responsive polyanion rotations?
- By what mechanism does the incorporation of lower-valent anions stabilize high-temperature, fast-ion-conducting phases?
- Are polyanion libration amplitudes significantly altered in low-temperature phases compared to room-temperature-stabilized high-temperature phases?

## 7 Conclusions and Outlook

This Perspective seeks to establish the structure-dynamics-property relationships that underpin the exceptional ion transport properties of the argyrodite family.

### 7.1 Interplay of Site Disorder and Dynamics

Anion site disorder remains a common, underlying design principle for ion transport in the argyrodites. In the context of this Perspective, site disorder appears to be strongly coupled to the complex dynamics in the argyrodite structure. The same electrostatic averaging across the 4*a* and 4*d* sites that gives rise to long-range Li^+^ percolation networks facilitates larger-amplitude polyanion rotations that take on a gate-like function to lower energetic barriers for intercage hopping. Concurrently, the onset of site disorder is associated with a noncompensatory drop in *E*_*A*_ and increase in σ_0_, contrary to Meyer-Neldel behavior. Further, σ_0_ scales exponentially with both site disorder and polyhedral displacement, implying that Δ*S*_*m*_ is modulated by the two factors. We hypothesize that site disorder promotes large amplitude oscillations of polyhedral units, which generate a broader distribution of vibrational environments and increase Δ*S*_*m*_. This points to an emerging relationship between site disorder, polyhedral rotations, and ion transport.

### 7.2 Phase Transitions

The aristotype-hettotype relationship is core to understanding the behavior of argyrodites. The adoption of an aristotypic *F*4̅3*m* average structure with ordered *MCh*_4_ tetrahedral units and delocalized mobile cation density is central to the argyrodite family. Low temperature hettotypes of the family represent minor modulations of the aristotype, generally with (a) localization of cations, (b) minor displacement of “free” anions, and (c) rotational modulations of the *MCh*_4_ tetrahedra. The hettotype-aristotype transition is best described as an order–disorder phase transition, where activation of polyhedral rotations leads to dynamic sampling of local hettotype arrangements that average to the high-symmetry aristotype at sufficiently long length scales. This behavior is captured by Bragg diffraction that can be well fit by the *F*4̅3*m* aristotype and local structure that is best described by hettotype structures or an *F*4̅3*m* structure with rotationally modulated polyhedra. This phase behavior is shared by the Na_3_PS_4_ family of fast ion conductors and can be stabilized by the incorporation of lower-valent anions; halide substitution in the argyrodites commonly stabilizes the aristotype at room temperature. We propose that this effect is due to halide substitution lowering the energy required to activate polyhedral rotational dynamics by weakening the electrostatic interactions between *MCh*_4_ units and “free” anion sites.

### 7.3 Future Directions

The richness of the dynamics and the interplay of static and dynamic disorder in these materials motivate several future directions for study.

- *Electrostatic Averaging vs Polyhedral Rotations:* At this stage, it is unclear whether electrostatic averaging due to site disorder is solely responsible for improved ion transport in argyrodites, or if activation of polyhedral dynamics through site disorder synergistically enhances ion transport. The causal relationships between site disorder, polyhedral dynamics, and improved ion transport should be interrogated to determine how polyhedral dynamics mediate between the other two.
- *Dynamic Disorder as a Defining Characteristic:* Our analysis of the argyrodite family predicts that dynamic disorder is inherent to the aristotype and therefore *all* aristotypic argyrodites will exhibit non-*F*4̅3*m* local symmetry. This behavior has been demonstrated in Li_6_PS_5_ *X* and a selection of Ag^+^-argyrodites, but further investigation to probe whether it is generalizable would be of great use to the field.
- *Aristotype Stabilization:* We hypothesize that halide substitution in the argyrodites stabilizes the aristotype by weakening electrostatic interactions between *MCh*_4_ units and “free” anions, thereby lowering the energy required for polyhedral rotations. This, in turn, is proposed to contribute to the observed phase transition behavior. Testing this hypothesis would provide insight as to how fast ion conducting phases may be stabilized at room temperature.

Argyrodites are compositionally diverse, structurally complex, and exhibit rich dynamic phenomena that are important for ion transport. A deeper, quantitative understanding of lattice dynamics, local polyanion rotations, and their coupling with mobile ions will not only clarify transport mechanisms in argyrodites but also inform broader design strategies for dynamic, fast-ion-conducting solids.
